# Tracking of Host Defenses and Phylogeny During the Radiation of Neotropical *Inga*-Feeding Sawflies (Hymenoptera; Argidae)

**DOI:** 10.3389/fpls.2018.01237

**Published:** 2018-08-23

**Authors:** María-José Endara, James A. Nicholls, Phyllis D. Coley, Dale L. Forrister, Gordon C. Younkin, Kyle G. Dexter, Catherine A. Kidner, R. T. Pennington, Graham N. Stone, Thomas A. Kursar

**Affiliations:** ^1^Department of Biology, The University of Utah, Salt Lake City, UT, United States; ^2^Centro de Investigación de la Biodiversidad y Cambio Climático (BioCamb) e Ingeniería en Biodiversidad y Recursos Genéticos, Facultad de Ciencias de Medio Ambiente, Universidad Tecnológica Indoamérica, Quito, Ecuador; ^3^Ashworth Laboratories, Institute of Evolutionary Biology, School of Biological Sciences, The University of Edinburgh, Edinburgh, United Kingdom; ^4^Smithsonian Tropical Research Institute, Panama City, Panama; ^5^Royal Botanic Garden Edinburgh, Edinburgh, United Kingdom; ^6^School of GeoSciences, The University of Edinburgh, Edinburgh, United Kingdom

**Keywords:** coevolution, defense traits, herbivores, host tracking, *Inga*, plant–insect interactions, sawflies, tropical rain forests

## Abstract

Coevolutionary theory has long predicted that the arms race between plants and herbivores is a major driver of host selection and diversification. At a local scale, plant defenses contribute significantly to the structure of herbivore assemblages and the high alpha diversity of plants in tropical rain forests. However, the general importance of plant defenses in host associations and divergence at regional scales remains unclear. Here, we examine the role of plant defensive traits and phylogeny in the evolution of host range and species divergence in leaf-feeding sawflies of the family Argidae associated with Neotropical trees in the genus *Inga* throughout the Amazon, the Guiana Shield and Panama. Our analyses show that the phylogenies of both the sawfly herbivores and their *Inga* hosts are congruent, and that sawflies radiated at approximately the same time, or more recently than their *Inga* hosts. Analyses controlling for phylogenetic effects show that the evolution of host use in the sawflies associated with *Inga* is better correlated with *Inga* chemistry than with *Inga* phylogeny, suggesting a pattern of delayed host tracking closely tied to host chemistry. Finally, phylogenetic analyses show that sister species of *Inga-*sawflies are dispersed across the Neotropics, suggesting a role for allopatric divergence and vicariance in *Inga* diversification. These results are consistent with the idea that host defensive traits play a key role not only in structuring the herbivore assemblages at a single site, but also in the processes shaping host association and species divergence at a regional scale.

## Introduction

Insect herbivores and their plant hosts dominate terrestrial biodiversity (Hunt et al., 2007), and the processes that drive their interaction and diversification remain an enduring focus of research in ecology and evolution (Futuyma and Agrawal, 2009; Janz, 2011; Hembry et al., 2014; Forbes et al., 2017; Nakadai, 2017). This is especially true in the tropics where most of the species occur. A central paradigm is that insect-plant associations have been shaped by arms race coevolution between plant defenses and insect countermeasures (Becerra, 1997; Becerra et al., 2009; Volf et al., 2018). Ehrlich and Raven (1964) observed that closely related plants are often attacked by closely related herbivores, a pattern they attributed to an ‘escape and radiate’ model, in which plant lineages diversify following evolutionary innovation of a key defense trait, and specialist herbivore lineages diversify across the plant radiation through evolution of a key countermeasure (Wheat et al., 2007). Where these traits are phylogenetically conserved in each lineage, we expect some degree of phylogenetic concordance between plant and herbivore lineages, resulting either from simultaneous co-diversification (Cruaud et al., 2012), or delayed herbivore colonization of an existing plant radiation (tracking of host resources; Janz, 2011). Thus, plant defenses play a prominent role in the evolution of host associations (Thompson, 1988), yet they are often not considered in studies of plant-herbivore diversification. Robust analyses require not only phylogenetic histories of both plants and herbivores, but also data on ecologically important traits such as plant defenses.

Although insect herbivores are expected to show evolutionary conservatism in host use (Ehrlich and Raven, 1964; Brooks and McLennan, 2002), many studies show herbivore shifts between distantly related hosts that disrupt any signature of codiversification. Some shifts are between hosts with similar chemical defenses for which herbivore countermeasures are to some extent preadapted, implying a process of host-resource tracking (Janz, 2011; Endara et al., 2017) or ecological fitting (Agosta and Klemens, 2008). Insect herbivores can also radiate across hosts with contrasting defensive traits through diversification of specialist host races, leading to ecological speciation (Nyman, 2010; Hardy and Otto, 2014). These alternative mechanisms of divergence without codiversification do not happen in isolation, and their impacts are expected to reflect the distributions of interacting lineages through time and space (Hoberg and Brooks, 2008; Züst et al., 2012; Calatayud et al., 2016). Assessing the contribution of these alternative mechanisms to observed patterns of interaction and diversity is thus a major challenge (Hembry et al., 2014; Russo et al., 2017). In particular, and with notable exceptions (e.g., Kursar et al., 2009; Wilson et al., 2012; Fine et al., 2013; Marquis et al., 2016; Salazar et al., 2016; Endara et al., 2017; Volf et al., 2018), we know little about the processes driving plant-herbivore diversification in the tropical rainforest areas that harbor most of terrestrial biodiversity (López-Carretero et al., 2018).

Here we explore the factors structuring associations between insect herbivores and neotropical trees in the genus *Inga*, a species-rich radiation that shows high local species richness and abundance in many habitats across the Neotropics, and which is characterized by high diversity of chemical, physical and developmental defenses against insect herbivores (Kursar et al., 2009). Previous analyses support a key role for *Inga* defensive chemistry in structuring lepidopteran herbivore assemblages at a single site (Endara et al., 2017), and non-random combinations of defensive traits across sites imply a role for herbivore avoidance in *Inga* community assembly (Kursar et al., 2009). It remains unclear, however, whether the same *Inga* traits structure herbivore associations in widely separated communities. Previous analyses have also found little phylogenetic pattern in *Inga* defenses (Kursar et al., 2009; Endara et al., 2015, 2017), and related lepidopteran herbivores attack *Inga*s with similar defenses, rather than those that are closely related (Endara et al., 2017). Our hypothesis is that herbivores have driven rapid diversification of defensive traits in *Inga*, with herbivore associations resulting from evolutionary tracking of similar defensive phenotypes (i.e., host-resource tracking) rather than cospeciation (Coley et al., 2018). Here we test this hypothesis using data for four regional communities that span the Amazon Basin, in Panama, Peru, Ecuador, and French Guiana. We focus on sawflies (Hymenoptera; Symphyta) in the superfamily Tenthredinoidea, shown in previous work in other regions of the world to be highly sensitive to (and often dependent on) toxic host plant chemistry (Petre et al., 2007; Boevé et al., 2013; Naya et al., 2016). Thus, they are an excellent candidate taxon in which to explore the impact of diversification in this key aspect of *Inga* defenses. We use novel data on *Inga-*sawfly associations and an analytical approach incorporating phylogenies for both lineages (Hadfield et al., 2014) and defense trait data to address the following questions: (i) Is there phylogenetic patterning in *Inga* defenses? (ii) Does *Inga* phylogeny (cospeciation) or defenses (resource tracking) best predict *Inga-*sawfly associations? (iii) Over what geographic scale have *Inga*-sawfly associations evolved? Are sister sawfly or *Inga* species commonly members of the same regional community, implying local, sympatric diversification? Or are sister taxa dispersed across the Neotropics, suggesting a role for allopatric divergence and vicariance in one or both trophic levels?

## Materials and Methods

### Sampling and Quantification of *Inga* Defensive Traits

We sampled 81 *Inga* species and 3 *Zygia* species (a sister clade of *Inga*) at four sites throughout the Amazon and Panama between July 2010 and September 2014: Panama (January-February 2010; Smithsonian Tropical Research Institute on Barro Colorado Island, 9.150°N, 79.850°W), French Guiana (July–August 2011 and 2012; Nouragues Station, 4.08°N, 52.683°W) Peru (July–October 2010 and 2011; Los Amigos Biological Station, Madre de Dios, 12.567°S, 70.100W) and Ecuador (July–September 2013 and 2014; Tiputini Biodiversity Station, 0.638°S. 76.150°W). In each location, we sampled expanding leaves of 0.5–4 m tall understory saplings. Host associations were recorded on c. 60 young leaf flushes per tree species. Sawfly larvae were found on 34 *Inga* and 2 *Zygia* species comprising from 1 to many gregarious larvae on a specific individual host plant (**Supplementary Table S2**).

We measured multiple defensive traits that capture the entire defensive profile of each species. These include developmental defenses (leaf expansion rate and chlorophyll content), biotic defenses (mean number of ants visiting the leaves and extra-floral nectary size) and chemical defenses. This set of defense traits was measured only on expanding leaves because more than 80% of the damage accrued during the leaf’s lifetime happens during the short period (1–3 weeks) of leaf expansion (Coley et al., 2018).

#### Developmental Defenses

Young leaves can expand rapidly, which shortens the window of vulnerability to herbivores, and they can delay chloroplast development, which reduces the impact of a given amount of damage (Kursar and Coley, 1992). Leaf expansion rate was determined as the percent increase in area per day for c. 13 individuals per species. Chloroplast development was measured as the chlorophyll content (mg dm^−2^) of leaves between 30 and 80% of full expansion for c. 30 individuals per species (Endara et al., 2017). Since these two traits are correlated, we treat them as a single defense.

#### Biotic Defenses

*Inga* leaves have extra-floral nectaries that produce nectar and attract protective ants only during the short period of leaf expansion. We quantified the diameter of these nectaries and the abundance of ants visiting them (# of ants per nectary) in c. 30 individuals per species.

#### Chemical Defenses

For chemical analyses, expanding leaves were dried in the field over silica at ambient temperature. Although *Inga* has little quantitative or qualitative induction of young leaf defenses (Bixenmann et al., 2016), samples used for chemical analyses were from plants without sawflies. The chemical defensive profile for each species was determined using metabolomics. Metabolites were extracted at the Coley/Kursar laboratory in the University of Utah in 44.3 mmol L^−1^ ammonium acetate, pH 4.8:acetonitrile (60:40, v/v) and analyzed following the protocol of Wiggins et al. (2016). Metabolites with intermediate polarity were analyzed by ultraperformance C18 liquid chromatography coupled to mass spectrometry (UPLC-MS) in negative mode. Raw data from the UPLC-MS analysis in MassLynx were converted to mzXML format using mzConvert (Chambers et al., 2012) and then processed for peak detection, peak alignment and peak filtering using the R package XCMS (Smith et al., 2006; Tautenhahn et al., 2008; Benton et al., 2010). These results were post-processed in the R package CAMERA to assign the various ions derived from one compound (termed ‘features’) to that compound (Kuhl et al., 2012), as detailed in **Appendix SII**. This analysis yields 2621 compounds from the 36 plant species. Purification and structure determination by 2-D NMR of several dozen compounds, as well as matching MS-MS spectra from our in-house database to the GNPS databases (Global Natural Products Social Molecular Networking)^1^ suggest that, for *Inga*, these compounds are mainly phenolics, saponins and amines. None are primary metabolites (**Supplementary Table S1**). All scripts from this study are deposited in github^2^.

Overexpression of the essential amino acid, L-tyrosine, ranges from 5 to 20% leaf DW (Dry Weight) in certain species of *Inga.* At these concentrations, it is highly toxic to non-adapted herbivores, and therefore functions as an important chemical defense (Lokvam et al., 2006). Because tyrosine is insoluble in our extraction buffer, tyrosine concentration as percent of leaf dry weight was determined separately following Lokvam et al. (2006, **Appendix SII**).

### Sawfly DNA Barcoding

Taxonomic resources are limited even for adult sawflies (Schmidt et al., 2017), and very few exist for morphological identification of neotropical sawfly larvae to species. We therefore adopted a DNA barcoding approach using sequences for a 645 base pair (bp) fragment of the mitochondrial gene cytochrome oxidase I (*COI*) (For DNA methods, see **Appendix SI**). Every individual was barcoded. For gregarious species, we sequenced a minimum of three in a group and in all cases these belonged to the same MOTU. Sequences were allocated to MOTUs (molecular operational taxonomic units) using two approaches: jMOTU v1.0.8 (Jones et al., 2011) and ABGD (Automatic Barcode Gap Discovery, Puillandre et al., 2012). jMOTU clusters sequences into MOTUs that differ by pre-defined numbers of bases; we examined divergence distances amongst sequences ranging from 1 to 65 bp, with a low BLAST identity filter of 97%. In the presence of a barcoding gap (Puillandre et al., 2012), a plot showing numbers of MOTUs as a function of sequences divergence should form a plateau, with no change in MOTU number across the divergence levels corresponding to the gap (Acs et al., 2010). ABGD defines MOTUs based upon prior values of within-species divergence, and assesses how MOTU number changes as within-species divergence increases. We used prior within-species divergence limits ranging from 0.3 to 6%, split into 30 steps. We used the K2P distance measure, with a transition to transversion ratio of 1.47, as estimated by jModeltest v2.1.7 (Darriba et al., 2012), and the default value of 1.5 for slope increase. Output from the recursive partitioning scheme was used, with the final number of MOTUs chosen at the point where the plot of MOTU versus intraspecific divergence leveled off. Both approaches gave highly concordant results.

Because mitochondrial haplotypes can be shared among species, and hence give misleading indications of species membership in sawflies (Prous et al., 2011; Schmidt et al., 2017) and more widely (Funk and Omland, 2003; Nicholls et al., 2012), we sequenced our candidate *COI* MOTUs for two nuclear loci, *wingless* (coding, 327 bp; *n* = 75 sequences) and *ITS2* (non-coding, 609 bp; *n* = 80; for molecular methods, see **Appendix SI**). Sampling incorporated all singleton MOTUs and 2–4 individuals of MOTUs with more extensive sampling. *COI* sequence data were highly effective in resolving relationships between sawfly samples with high posterior probability (**Supplementary Figure S1**). The first barcoding gap using jMOTU was apparently at 7–10 bp (1–1.5% divergence), identifying 42 MOTUs. These were highly concordant with 40 MOTUs identified by ABGD for sequence divergence from 1.03 to 1.41% (**Supplementary Figure S2**), the only difference being that jMOTU split two of the 40 ABGD MOTUs into two. Relationships between MOTUs identified using *COI* data were highly concordant with those based on nuclear *ITS2* and *wingless* (**Supplementary Figures S3**, **S4**). Our final sawfly MOTU definitions (*n* = 41) incorporated information from both mitochondrial and nuclear data, with *COI* MOTUs retained if at last one nuclear gene showed the same clustering of individuals. Sawfly MOTUs were allocated to candidate taxonomic families by querying each against voucher sequences in the Barcoding of Life BOLDSYSTEMS database^3^.

Gene trees for each of the three loci were generated using MrBayes *v*3.2.2 (Ronquist et al., 2012). Based on relative numbers of variable sites at each codon position, *wingless* was treated as a single partition while *COI* was partitioned between codon positions 1 + 2, and position 3. As a non-coding locus, *ITS2* was treated as a single partition. We used the closest available substitution model in MrBayes as per the recommendation provided by *jModeltest* (Guindon and Gascuel, 2003; Darriba et al., 2012), as follows: *COI*(1,2), GTR+I+G; *COI*(3), GTR+G; *wingless*, GTR+I+G; *ITS2*, GTR+G. MrBayes analyses were run for 20 million generations for *ITS2* and *wingless*, sampling every 2500 generations, with a burn-in of 16 million generations. The analysis for *COI* was run for 40 million generations to achieve convergence, sampling every 5000 generations, with a burn-in of 32 million generations. Likelihood comparisons showed a relaxed IGR clock model to be better supported than either no clock or a strict clock for all loci.

### Phylogenetic Relationships Among Sawfly MOTUs

We determined phylogenetic relationships for our MOTUs at two levels. To place our *Inga-*feeding sawfly MOTUs in a wider phylogenetic context, we carried out additional phylogenetic analyses using data for *COI* and an additional coding nuclear locus, *PGD* (496 bp). *PGD* data provided high resolution in a previous wide-ranging phylogenetic analysis of sawflies (Malm and Nyman, 2015), and allow us to place our MOTUs within this taxonomic framework. Analyses for each gene incorporated data on sawfly species from recent phylogenetic (Schulmeister et al., 2002; Malm and Nyman, 2015) and barcoding (Hartsough et al., 2007; Schmidt et al., 2017) surveys of sawflies. These analyses identified related taxa on the basis of nearest matches identified from BOLD. The taxa from the surveys that we added to our analysis comprised 11 species in the family Tenthredinidae, 9 in the family Pergidae and 34 in the family Argidae, none of which are neotropical. We also included a similar number of sequences for neotropical taxa, and the only available *COI* voucher sequence for an *Inga*-feeding sawfly, a specimen of *Ptenos leucopoda* (Argidae) sampled from *Inga oerstediana* (and also recorded from *I. vera*) in Costa Rica (Smith et al., 2013). Metadata and Genbank accession numbers for these reference sequences are provided in **Supplementary Table S3**. We constructed gene trees for each locus using MrBayes, using the closest available substitution model to that identified as appropriate using *jModeltest* (Guindon and Gascuel, 2003; Nylander, 2004; Darriba et al., 2012). Based on relative numbers of variable sites at each codon position, *PGD* data were modeled in two partitions, 1 + 2, and 3, each with a GTR+I+G model, while *COI* was divided into three partitions by codon, each with a GTR+I+G model. For each gene we assumed a relaxed clock, with a birth-death speciation model. To provide an order of magnitude age for *Inga*-associated sawfly lineages, we calibrated the *COI* tree using two alternative estimates: the Brower rate estimate of 0.0115 substitutions per million years (Brower, 1994) and the higher rate of 0.0177 derived by Papadopoulou et al. (2010).

For analysis of evolutionary dynamics in sawfly *Inga* trophic associations, we generated an overall species (MOTU) tree using data for all four loci (*COI, ITS2, PDG, wingless;* 2077 bp) for the 39 MOTUs identified as putative Argidae using the Bayesian ^∗^BEAST algorithm (Heled and Drummond, 2010) within BEAST v2.4.1 (Drummond et al., 2012). The ^∗^BEAST model used 5 partitions with the following substitution models: *COI* (codon positions 1,2), TN+I+G; *COI* (codon position 3), TN+G; *wingless*, GTR+I+G; *ITS2*, GTR+G; *PGD*, GTR+I+G. We used a Yule speciation model, and compared likelihood support for each combination of relaxed *versus* strict clock models and constant *versus* linearly changing population size. This approach supported a constant population size and an independent relaxed lognormal clock for each partition. We carried out two runs of the ^∗^BEAST analysis, with outputs combined in Logcombiner, part of the BEAST suite (Drummond et al., 2012). Each run was for 500 million generations, sampling every 62500 generations, with a burn-in of 300 million generations. Analysis of run diagnostics in Tracer v1.6 (Rambaut and Drummond, 2007) showed all parameters to have an effective sample size of > 100.

### Generation of an *Inga* Species Tree

We constructed a species tree for 77 *Inga* accessions representing the taxa from which sawflies were collected, using data for ten coding nuclear loci previously identified as being phylogenetically informative in a wider study of *Inga* phylogenomics (Nicholls et al., 2015) (**Supplementary Table S4**). Aligned sequences for each locus in all *Inga* specimens are available from the Dryad Digital Repository^4^. The ten loci ranged in length from 272 to 2767 bp, with 9–14.7% of sites variable, and spanned a total of 16,125 bp (**Supplementary Table S4**). All ten loci were sequenced in all 77 *Inga* accessions. We co-estimated gene tree topologies and an overall species tree topology using ^∗^BEAST, as described above. We used the substitution model previously identified for each locus by Nicholls et al. (2015) (**Supplementary Table S4**). We specified a Yule speciation model and assumed a constant population size. We selected a relaxed lognormal clock over a strict clock model based on very high Bayes factor support (574, estimated as 2Ln harmonic mean likelihood) following criteria in Kass and Raftery (1995). Our analyses ran for 500 million generations, sampled every 62,500 generations, with a burn-in of 50 million generations. Analysis of run diagnostics in Tracer v1.6 (Rambaut and Drummond, 2007) showed all parameters to have an effective sample size of > 100.

### Data Analysis

#### Estimation of Sampling Effort

Sawfly MOTU accumulation curves were generated in the Vegan R package using sampling over *Inga* species [specaccum(data, “random”)] and sampling over sawfly individuals [specaccum(data, method = “rarefaction”)]. The “random” method finds the mean accumulation curve and its standard deviation from random permutations of the data. The “rarefaction” method finds the expected species richness and its standard deviation by sampling individuals instead of sites. It achieves this by applying function “rarefy” with number of individuals corresponding to average number of individuals per *Inga* species – which for our data is 1286 sawflies/34 plant taxa = 38 individuals.

#### Chemical Similarity Between Species of *Inga*

We analyzed data for phenolics and saponins separately. Saponins were defined as all compounds with chromatographic retention time > 18 min and *m/z* > 580 for the precursor ion, with the remainder classified as phenolics. For several *Inga* species, early eluting compounds have been purified and their structures elucidated by 2D-NMR (J. Lokvam, unpublished). This shows that the bulk of early eluting compounds are phenolics. For about 10 species, the late-eluting fraction was separated from phenolics, hydrolyzed to remove sugars, the triterpene aglycons isolated and their structures elucidated by 2D-NMR (J. Lokvam, unpublished). This work indicates that the bulk of the late-eluting compounds are saponins. Certainly, we cannot rule out that some peaks may belong to other classes. Compounds that are shared across species were matched based on *m/z* (mass to charge ratio) and retention time. Because many compounds, 1097 out of 2621, are found in only one species, we also quantified species similarity based on the structural similarity of unshared compounds. This matters because unshared compounds are typically treated as having zero relationship even though they may have significant structural similarity. In metabolomics, molecules can be identified based on whether the MS fragmentation pattern (MS/MS spectrum) of an unknown matches spectra in curated databases. A limitation is that these databases include few secondary metabolites, providing little opportunity to quantify the structural relatedness of similar molecules. A recent advance is to quantify the similarity of the MS/MS spectra of a large number of molecules. These data generate a network using the online workflow at the Global Natural Products Social Molecular Networking site (GNPS)^5^. In the resulting network, each node or circle represents a unique compound, with edges (lines) connecting nodes based on structural similarity. Each pair of compounds is assigned a structural similarity score ranging from 0 (completely dissimilar) to 1 (identical) based on the similarity of their MS/MS fragmentation spectra (Watrous et al., 2012). To accomplish this, we obtained as many MS/MS spectra as possible, for 1925 out of our 2621 study compounds. See **Appendix SII** for MS/MS methods and calculation of the chemical similarity of species from molecular networks.

We constructed a dendrogram of chemical similarity between species by fitting a hierarchical clustering model to the equally weighted chemical similarity matrix with 10,000 permutations using the R package PCVLUST (Suzuki and Shimodaira, 2014). For more details see **Appendix SII**.

Because there are many possible equations and data transformations for calculating species similarity scores, we compared several of these alternatives to lepidopteran dietary preferences following Endara et al. (2017). These analyses validated our method (**Appendix SII**).

#### Relationship Between Plant Traits and Phylogenetic Signal

Phylogenetic signal was evaluated for continuous host defensive trait data (developmental and biotic defenses), and for the principal coordinates of the chemistry similarity matrix using Blomberg’s *K* (Blomberg et al., 2003). *K* is close to zero for traits lacking phylogenetic signal, but close to one for traits whose values through the phylogeny match expectations under a Brownian model of evolution. We used the function *phylosig* in the R package *phytools* v.0.6-44 (Revell, 2017).

### Analysis of Herbivore–Host Plant Associations

Due to the gregarious habit of sawflies, we use incidence data (presence–absence) for analyses of host associations. Thus, if a specific MOTU was associated with a specific *Inga* host plant in several sampling events on the same plant, it would have been counted only once. To determine the extent to which host phylogeny and/or host defenses structure the associations between sawflies and their hosts, we used maximum likelihood to model the probability of sawfly occurrence *(p)* using a binomial distribution with the number of trials equal to the total number of herbivore species associated with each *Inga* species. These analyses included all *Inga* species, even those on which sawflies were never found, so that we could determine which *Inga* traits predict an association with any sawfly MOTU. We fitted models that incorporated only the intercept, and the effects of one or more *Inga* defensive traits and the principal coordinates of the phylogenetic distance matrix and the chemical similarity matrix using the R packages *bbmle* v.1.0.20 (Bolker, 2017) and *emdbook* v.1.3.9 (Bolker, 2016). For these analyses, we used the whole *Inga* phylogeny (unpublished *Inga* phylogeny, Nicholls et al., unpublished). The models were run using sampling effort as a covariate (number of leaf flushes searched per *Inga* species). We performed model comparison based on Akaike Information Criterion for small sample sizes (AICc).

Evolutionary interactions between sawflies and *Inga* hosts were determined using a Bayesian approach with generalized linear mixed-effects models (GLMM) in the R library *MCMCglmm* (Hadfield and Nakagawa, 2010; Hadfield, 2017). We performed these analyses only with those *Inga* species that are associated with sawflies. Following Hadfield et al. (2014), we partitioned variance in the sawfly incidence data per *Inga* host into the effects of the phylogenetic histories of plants and herbivores, whether in isolation (termed evolutionary effects by Hadfield et al., 2014) or as interactions (a coevolutionary effect), and chemical similarity between *Inga* hosts (a defense effect). This model approach also allows the estimation of other factors, where interactions have evolved independently of the phylogenies and *Inga* chemistry similarity. The magnitude of the effect for each term is determined by the magnitude of the variance. Following Hadfield et al. (2014), the first term in the model captures the effect of the geographic region information (here termed Geographical region). The second term determines the contribution of the main effect of the sawfly phylogeny to the covariance and captures the variation in host range explained by the phylogeny (Phylogenetic main effect for sawflies). The third term is the contribution of the main effect of *Inga* chemistry to the covariance and captures the variation in sawfly species richness explained by chemical similarity between *Inga* hosts (Defensive main effect for *Inga* hosts). The fourth term is the contribution of the main effect of *Inga* phylogeny to the covariance and captures the variation in sawfly species richness explained by the phylogeny of the *Inga* hosts (Phylogenetic main effect for *Inga* hosts). The fifth term captures the degree to which related *Inga* have similar sawfly assemblages irrespective of sawfly phylogeny (Phylogenetic *Inga* evolutionary effect). The sixth term captures the degree to which species that are similar in chemistry have similar sawfly assemblages irrespective of sawfly phylogeny (*Inga* defense interaction). The seventh term captures the degree to which related sawflies have similar *Inga* hosts assemblages irrespective of *Inga* phylogeny (Phylogenetic parasite evolutionary effect). The eighth term is the contribution of the coevolutionary interaction to the covariance and captures the degree to which related sawflies feed on related *Inga* (Coevolutionary effect). The ninth term is the contribution of the interaction between *Inga* chemistry and sawfly phylogeny and captures the degree to which related sawflies feed on *Inga* that are similar in chemistry (Defense tracking effect). The last three terms capture interspecific variation in host range (Main effect for sawflies), interspecific variation in sawfly species richness (Main effect for *Inga* hosts) and associations between specific *Inga* hosts and sawflies species (Interaction effect) not due to phylogeny or chemistry.

Phylogeny and chemistry were incorporated into the model as variance-covariance matrices of relatedness and similarity, respectively, in the random effect structure of the generalized linear mixed effect model. We compared models that included site effects (analyses at large spatial scales, as a random factor) and which controlled for sampling effort (as a fixed factor), with models that ignored between-site patterns (hence, analyses at small spatial scales) and sampling effort completely. For the analyses, parameter-expanded priors were used for all variance components following Hadfield et al. (2014). The chain was run for 500,000 iterations with a burn-in of 50,000 and a thinning interval of 450. Because the response variable was incidence data, a Bernoulli error distribution was applied. Models were fitted using the R package *MCMCglmm* v.2.23 (Hadfield, 2017).

Correlations between sawfly phylogenetic relationships with host plant phylogenetic relationships and with host plant chemistry were explored using the function *parafit* (Legendre et al., 2002) in the R package *Ape* v.5.0 (Paradis et al., 2004). We used the global test in *parafit* to test the null hypotheses that (i) the evolution of sawflies and *Inga*, as revealed by the two phylogenetic trees and their trophic associations, has been independent; and (ii) by substituting the *Inga* chemogram for the *Inga* phylogeny that sawfly diversification has been independent of host plant chemistry. Pairwise patristic distances were extracted between sawfly MOTUs from the 4-locus Argidae species tree, and between their corresponding *Inga* host plants from the 10-locus species tree and *Inga* chemogram using the *cophenetic.phylo* command in *Ape. Parafit* analyses used 9999 permutations. Matches between the sawfly phylogeny and each of the *Inga* phylogeny and chemogram were optimized using the function *cophylo* in the R package *phytools* (Revell, 2017).

Visualization of the *Inga*-sawfly associations in phylogenetic space was performed using a Principal Component Analysis. Using the function *phylomorphospace* in the R package *phytools* (Revell, 2017), phylogenetic relationships between sawfly MOTUs was mapped onto *Inga* phylospace. For this analysis, we use the whole *Inga* phylogeny (unpublished *Inga* phylogeny, Nicholls et al., unpublished).

## Results

### *Inga* Sawflies Are a Diverse Monophyletic Radiation of Specialist Herbivores Within the Family Argidae

**FIGURE 1:**
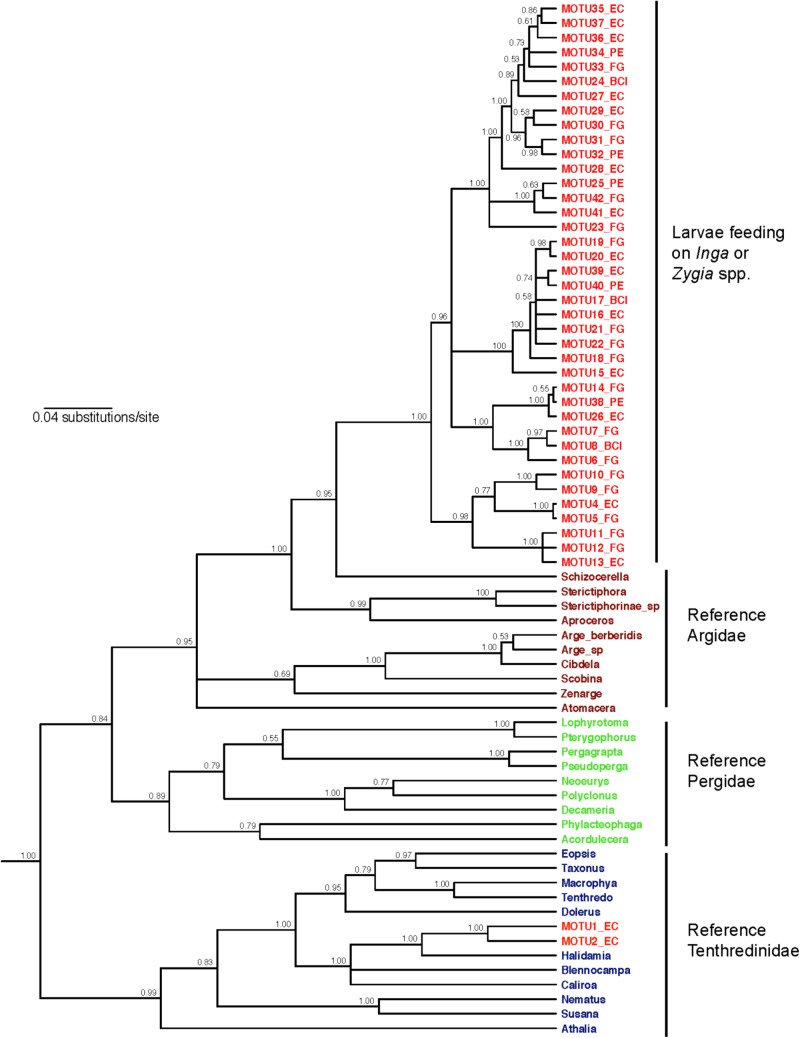
Phylogenetic relationships for the gene *PGD* among the *Inga*-feeding sawfly MOTUs and a panel of voucher sequences for sawflies in the families Argidae, Pergidae (sister group to Argidae; Malm and Nyman, 2015) and Tenthredinidae. The tree shown is a majority-rule consensus tree constructed in MrBayes, using substitutions modeled as GTR+I+G for 1st and 2nd codon positions combined, and GTR+I+G for 3rd positions. We used a relaxed clock, with a birth-death speciation model. Numbers at nodes indicate posterior probability. Taxon labels are colored by sampling source: red MOTU numbers are larvae found feeding on *Inga* or *Zygia*, while other colors indicate reference sequences for adult Argidae, Pergidae, and Tenthredinidae.

Our *COI* barcoding approach identified 41 MOTUs for sawflies feeding on *Inga* and *Zygia* host plants (**Supplementary Figure S1**), differing by 7–10 bp (1–1.5% divergence). Each sawfly MOTU attacked a very narrow range of 1–2 host *Inga* species, and each *Inga* species only hosted a small number of sawfly MOTUs. This pattern is consistent with the MOTU accumulation curve across sampled sawfly individuals, which suggested that adding more *Inga* taxa to the sampling would only add more specialist sawflies (e.g., sawfly MOTU accumulation curve across *Inga* species rise sharply, **Supplementary Figure S5**). In addition, because the sawfly MOTU accumulation curve across sampled sawfly individuals is asymptotic, this indicates that a more extensive sampling would not yield many additional *Inga*-sawfly interactions (**Supplementary Figure S5**). Thirty-nine MOTUs were identified by BOLD query as likely members of the family Argidae, while the remaining two were most similar to sequences for species in the family Tenthredinidae (**Supplementary Table S2**). Phylogenetic analysis showed that the 39 putative Argidae comprise a well-supported monophyletic clade within this family for the nuclear PGD locus (**Figure 1**; clade posterior probability = 1.0) and also for the more extensive taxon set sequenced for mitochondrial *COI* (**Supplementary Figure S6**). The remaining two MOTUs were placed within a strongly supported clade of voucher sequences for the family Tenthredinidae (**Figure 1**; PP = 0.99). Calibrations of the mutation rate for *CO1* estimate the median age of the common ancestor of this Argidae clade at 6.27 (95% confidence interval 4.78–7.93) million years using the Brower (1994) estimate and 5.31 (4.05–6.72) million years using the Papadopoulou et al. (2010) estimate.

### Sawflies Feed on a Chemically Distinct Subset of Available *Inga* Hosts

**FIGURE 2:**
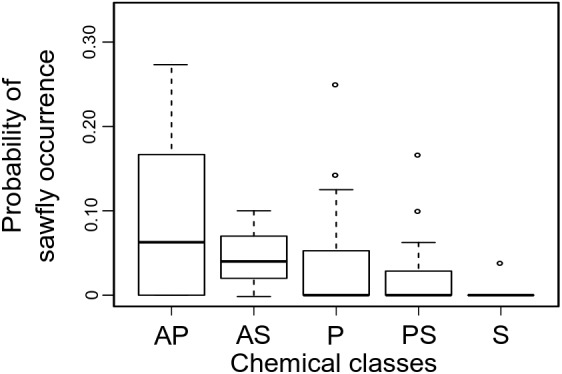
Sawfly occurrence for each *Inga* host chemotype. Shown is the range and distribution of proportion of occurrence of sawfly MOTUs per *Inga* chemotype. Chemistry is represented by the main chemical classes found in *Inga.* AP, Amines + phenolics; AS, Amines + saponins; P, Phenolics; PS, Phenolics + saponins; S, Saponins. The box shows the median and the 25%- and 75% percentiles. The whiskers are the 1.5 × interquartile range; outliers are drawn as individual points.

We investigated the specific role of each host trait in predicting sawfly *Inga* associations in analyses including joint-absence information (i.e., analyses including observations where sawflies were never collected on certain species of *Inga*). We found that similarity in chemical defenses among *Inga* hosts was the most important predictor for the occurrence of sawflies in general [proportional odds estimate for PCO1 = 0.26, (95% CI = 1.3 – 0.04), proportional odds estimate for PCO2 = 0.13, (95% CI = 0.95 – 0.02)]. Specifically, sawflies as a group prefer hosts that are defended by amine metabolites [proportional odds estimate for the presence of amines = 1.52, 95% CI (9.89 to 0.41), **Figure 2**], while the probability of occurrence of sawflies decreases with the presence of saponins [proportional odds estimate for the presence of saponins = 0.18, 95% CI (1.99 to 0.008), **Figure 2**].

### Closely Related *Inga* Hosts Fed on by Sawflies Are Similar in Chemical and Developmental Defenses

**FIGURE 3:**
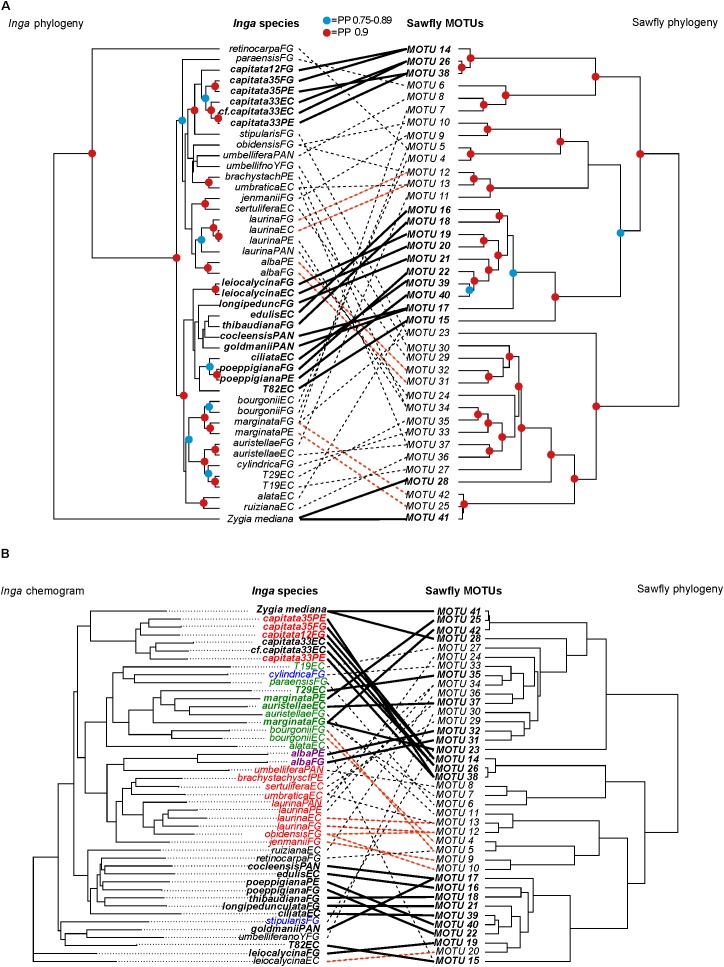
Patterns of diversification in Argidae sawfly MOTUs, mapped against **(A)** the phylogeny of their *Inga* food plants, and **(B)** a phenogram of host chemical defenses (‘chemogram’). The match between topologies in each case was optimized using the *cophylo* command in the R *Phytools* package. The sawfly and *Inga* phylogenies are maximum clade credibility species trees produced from multilocus analyses in ^∗^Beast, for four and ten loci respectively **(A,B)**. Links and taxon names highlighted in bold are identified as individually significant in *parafit* analyses (see **Supplementary Tables S4**, **S5**), while links highlighted in red show additional examples of closely related sawflies feeding on geographically separated populations of the same host plant species. The remaining links are indicated as dashed lines. The geographic location of *Inga* populations is indicated in the taxon labels as follows: EC, Ecuador; FG, French Guiana; PAN, Panama; PE, Peru. Colored symbols at nodes on phylogenies indicate posterior probability (PP) support: red = PP from 0.9 to 1.0, blue = PP from 0.75 to 0.89. *Inga* species in **(B)** are color-coded by chemotype: black (phenolics), red (phenolics + amines), green (phenolics + saponins), blue (saponins) and purple (saponins + amines).

For the *Inga* that were fed upon by sawflies, we quantified chemical similarity between species based on the similarity of chemical structure and relative abundance of compounds. We found that closely related *Inga* species and geographically separated populations of the same *Inga* species tend to have similar chemical defenses. Principal coordinates of the chemistry similarity matrix showed phylogenetic signal (PCO1 *K* = 0.71, p = 0.0002; PCO2 *K* = 1, *p* = 0.0001, **Table 1**). For example, lineages from the *Inga capitata* species complex (**Figure 3A**, left-hand phylogeny) share a series of tyramine gallates and quinic acid gallates. Similarly, the clade containing *Inga edulis, Inga poeppigiana*, *Inga ruiziana* and *Inga thibaudiana* share similar chemistry based on gallocatechin/epigallocatechin gallates. However, we find examples of closely related taxa with contrasting chemistry, a typical pattern for the genus as a whole (Kursar et al., 2009). For instance, *Inga umbellifera_no_Y* in French Guiana lacks overexpression of tyrosine in expanding leaves, whereas its sister species, *I. umbellifera* from Panama, contains 10.1% of leaf dry mass as tyrosine.

**Table 1:** Measure of phylogenetic signal for each *Inga* defensive trait and the principal coordinates of the chemistry similarity matrix (PCO) using Blomberg’s *K.*

Defensive traits	*K statistic*	*P* (reps = 9999)
Chemistry PCO1 (39%)	0.71	0.0002
Chemistry PCO2 (17%)	1	0.0001
Leaf expansion rate	0.37	0.05
Chlorophyll content	0.49	0.01
Ant number	0.12	0.58
Extra-floral nectary size	0.09	0.8

*For PCO components, values in parentheses represent the percentage of variation explained by each component.*

Developmental defenses of *Inga* species fed on by sawflies showed a similar pattern to chemistry. Leaf expansion rate and chlorophyll content showed weak phylogenetic signal (leaf expansion rate *K* = 0.37, *p* = 0.05, chlorophyll content *K* = 0.49, *p* = 0.001). In contrast, biotic defenses were divergent among close relatives in *Inga* that are sawfly hosts, with no evidence for phylogenetic conservatism in ant visitation and extra-floral nectary size (**Table 1**).

### Chemically Similar *Inga* Hosts Are Attacked by Similar Sets of Sawflies

Evolutionary interactions between sawflies and their *Inga* hosts were tested using a four-locus phylogeny for Argidae sawfly MOTUs and a ten-locus phylogeny for their *Inga* food plants. Because only chemistry was selected as an important predictor for sawfly *Inga* associations, the following analyses were performed without the other host defensive traits. Phylogenies for both groups were well resolved, with strong posterior support at many nodes (**Figure 3A**).

*Parafit* analysis revealed a significant signature of codiversification between these two groups (global correlation, *p* = 0.015). The 19 sawfly-*Inga* interactions contributing most strongly to this pattern are concentrated in two sawfly and *Inga* clades (**Figure 3A**), and include closely related sawfly MOTUs that feed on geographically separated populations of the same species of *Inga* (see highlighted links in **Figure 3A** for sawflies feeding on *Inga alba, Inga capitata, Inga laurina, Inga leiocalycina, Inga marginata*, and *Inga poeppigiana*). However, there are also multiple examples of a single sawfly MOTU that feeds on phylogenetically divergent host plants (e.g., *Inga auristellae* and *Inga umbratica* attacked by MOTU 37 in Ecuador, *Inga stipularis* and *Inga marginata* attacked by MOTU23 in French Guiana, *Inga retinocarpaFG* and *Inga bourgoniiFG* attacked by MOTU 5), and divergent *Inga* hosts attacked by closely related sawfly MOTUs (e.g., *Inga umbraticaEC* and *Inga auristellaeFG* attacked by MOTUs 11 and 13). A single *Inga* species can also be attacked by phylogenetically divergent sawfly MOTUs (e.g., *Inga marginata*FG attacked by sawfly MOTUs 7, 42 and 23, and *Inga umbratica* attacked by MOTUs 13 and 37).

There is a much stronger correlation between the sawfly phylogeny and *Inga* chemistry (global correlation, *p* = 0.001) (**Figure 3B**). Many of the links contributing to this pattern (18 of 25 interactions, **Supplementary Table S5**) are the same as those contributing to the correlation between the *Inga* and sawfly phylogenies. There are many examples of closely related sawfly MOTUs attacking chemically similar *Inga* taxa (**Figure 3B**). In some cases, the two chemically similar *Inga* species are not closely related phylogenetically. For example, sawfly MOTU 12 attacks both *Inga laurina* and *Inga obidensis* in French Guiana. These two host plants have similar chemistry (**Figure 3B**), but are quite divergent phylogenetically (**Figure 3A**). There are four examples of the same sawfly MOTU attacking two hosts that are very divergent chemically (sawfly MOTU 15 attacking both *Inga* T82, *Inga alata* and MOTU 37 attacking *Inga auristellae*, *Inga umbratica* in Ecuador; MOTU 5 attacking *Inga retinocarpa* and *Inga bourgoniii* and MOTU 23 attacking *Inga marginata* and *Inga stipularis* in French Guiana) (**Figure 3B**).

In agreement with *Parafit* analyses, our *MCMCglmm* evolutionary models incorporating phylogenetic and chemical effects showed that the defense interaction term contributed the greatest variation to the sawfly incidence data, suggesting that the association between sawflies and *Inga* hosts is mainly due to chemistry (*Inga* defense interaction term in **Table 2**). The defense interaction term is the only term whose lower confidence limits exclude zero in any model, and this is true for all four models in **Table 2**. Chemically similar *Inga* species are attacked by related sets of sawfly MOTUs, having taken sawfly phylogeny into account. This was true in models with and without between-site information and sampling effort (**Table 2**). At large spatial scales (models with between-site information), coevolutionary and defense tracking effects were moderately large indicating that closely related sawflies are feeding on closely related *Inga*, which also are similar in chemistry (**Table 2**). However, when the models were fitted without controlling for sampling size and at small spatial scales (without between-site information), both the coevolutionary effect and the defense tracking effect decreased. Geographic region has a large effect in all models (**Table 2**). In some cases, closely related species of sawflies are separated by geography but feed on the same species of *Inga.* For example, MOTU 31 attacks *Inga alba* in Peru, and its sister species MOTU 32 is associated with *Inga alba* in French Guiana (**Figures 3A,B**). MOTU 19 is associated with *Inga leiocalycina* in French Guiana and the sister lineage, MOTU 20, is associated with *Inga leiocalycina* in Ecuador (**Figures 3A,B**). These observations are most consistent with an allopatric mode of sawfly speciation, suggesting that biogeography is an important component in sawfly *Inga* associations.

**Table 2:** Proportion of variation in sawfly incidence data attributed to phylogenetic and defensive terms.

	Including geographical region information		Without geographical region information	
	Controlling for sampling effort	Not controlling for sampling effort	Controlling for sampling effort	Not controlling for sampling effort
Geographical region	0.163 (0.000–0.591)	0.165 (0.000–0.596)	0.214 (0.000–0.783)	0.236 (0.000–0.955)
Phylogenetic main effect for sawflies	0.009 (0.000–0.042)	0.003 (0.000–0.015)	0.010 (0.000–0.04)	0.010 (0.000–0.041)
Phylogenetic main effect for *Inga* hosts	0.005 (0.000–0.021)	0.012 (0.000–0.005)	0.006 (0.000–0.029)	0.006 (0.000–0.024)
Defense main effect for *Inga* hosts	0.018 (0.000–0.074)	0.015 (0.000–0.055)	0.011 (0.000–0.449)	0.009 (0.000–0.036)
*Inga* hosts evolutionary interaction	0.009 (0.000–0.038)	0.007 (0.000–0.03)	0.016 (0.000–0.063)	0.020 (0.000–0.065)
*Inga* defense interaction	0.537 (0.091–1.009)	0.546 (0.104–0.967)	0.663 (0.188–1.238)	0.650 (0.236–1.039)
Sawfly evolutionary interaction	0.019 (0.000–0.251)	0.019 (0.000–0.071)	0.016 (0.000–0.066)	0.015 (0.000–0.057)
Coevolutionary interaction	0.065 (0.000–0.251)	0.058 (0.000–0.293)	0.015 (0.000–0.10)	0.010 (0.000–0.045)
Defense tracking interaction	0.065 (0.000–0.250)	0.051 (0.000–0.161)	0.026 (0.000–0.10)	0.020 (0.000–0.078)
Main effect for sawflies	0.005 (0.000–0.021)	0.007 (0.000–0.02)	0.005 (0.000–0.023)	0.008 (0.000–0.034)
Main effect for *Inga* hosts	0.006 (0.000–0.026)	0.007 (0.000–0.028)	0.006 (0.000–0.028)	0.004 (0.000–0.015)
Interaction effect	0.063 (0.000–0.241)	0.069 (0.000–0.264)		

*Columns contain the posterior modes (with 95% confidence intervals in parentheses) for the estimates. See Materials and Methods for a description of each term.*

**FIGURE 4:**
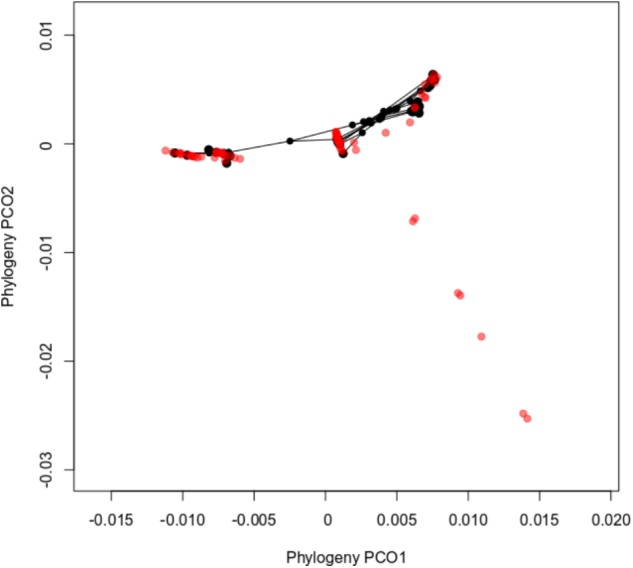
Principal coordinates analyses plots of sawfly MOTUS-*Inga* hosts associations in terms of *Inga* phylogeny. Each point in the figure represents an *Inga* species, including those on which sawflies were never found, colored red. *Inga* that are associated with sawflies are colored in black. Points that are close together in the phylogenetic ordination diagram indicate closely related Inga species. Lines connecting the points represent sawfly phylogenetic relationships. *Inga* species that are located at and below the coordinate −0.01 in the y axis represent basal branches in the *Inga* phylogeny.

The ordination diagram of the sawfly *Inga* associations in phylogenetic space (**Figure 4**) supported these findings by clustering sawfly MOTUS associated with *Inga* hosts that are closely related. This graph also shows the level of specialization for sawflies. The portion of *Inga* phylogenetic space towards the bottom right has seven species upon which we did not find any sawflies. These belong to early-diverging lineages of *Inga*. In fact, the sampled sawfly species feed entirely on one clade of *Inga*, albeit a clade that encompasses the large majority of *Inga* species.

## Discussion

### Sawfly Barcoding

Work on tropical plant-herbivore associations has long been hampered by lack of taxonomic resources. DNA barcoding is well established as a major tool in circumventing this taxonomic impediment in species-rich tropical ecosystems (Janzen et al., 2005; Miller et al., 2016). Our barcoding of sawfly larvae has generated host plant association data for 41 *Inga* or *Zygia*-feeding MOTUs, and represents a substantial extension to what is known for neotropical sawflies. Forty of the full set of 41 MOTUs (38 of the 39 putative Argidae MOTUs) are novel. Two putative Argidae specimens from Barro Colorado, Panama, showed a 99% match to a voucher sequence for the argid species *Ptenos leucopoda*, described from Guanacaste, Costa Rica, and are probably members of this species. Twenty-two other individuals in eight MOTUs showed ≥ 90% sequence similarity to voucher sequence for species in the Argidae genus *Ptenos*, and are also probably members of this genus.

Sawfly faunas in many tropical regions of the world remain relatively understudied, and even where adults have been sampled the larval foodplants of most species remain unknown. As an example, the genus *Ptenos*, to which some of our *Inga-*sampled sawflies certainly belong, contains around 31 species from the southwestern United States to Argentina, but to our knowledge, published food plant associations are only known for one species, *P. leucopoda* (Smith et al., 2013). Pairing of adults and larval stages is a major benefit of DNA barcoding (e.g., Stone et al., 2008) – but few voucher barcode sequences for identified adults exist for many groups of sawflies. For example, Schmidt et al. *(*2017) reported BOLD reference barcode sequences for only 49 of the 918 known Argidae species worldwide. Only one of our specimens showed a high match to an identified voucher, for *Ptenos leucopoda* from Costa Rica. While sequence match places the other 40 MOTUs confidently within the families Argidae (*n* = 38) and Tenthredinidae (*n* = 2), their species status remains to be determined. The sequence divergence threshold we have used, at 1.5%, is slightly lower than the 2% applied by Schmidt et al. (2017) for the same sequence region in their Europe-focused barcode study of sawflies. However, Schmidt et al. (2017) found sequences for 13 of 49 Argidae voucher taxa to differ by less than 2%, suggesting that our empirically determined lower threshold is appropriate for this group.

### *Inga-*Sawfly Evolutionary Associations

Our results extend Ehrlich and Raven’s main prediction that closely related plants are associated with closely related herbivores (Ehrlich and Raven, 1964). Colonization of *Inga* by sawflies seems to have been restricted to two events: (1) once by the ancestor of the *Inga*-associated Argidae clade, and (2) once by the common ancestor of the two *Inga*-associated Tenthredinidae MOTUs (**Figure 1**). Here we focus on Argidae. Given the high phylogenetic conservatism for chemical defenses in the species of *Inga* associated with sawflies (**Table 1**), we would predict high topological congruence between *Inga* and sawfly phylogenies. Evolutionary analysis suggested a significant congruence between both topologies (**Figure 3A**). This result is further supported by the monophyly of the argid sawflies associated with *Inga.* Most *Inga* and *Zygia*-associated sawflies belong to a single clade that can be confidently placed in the family Argidae with reference to identified reference material – including a sequence match with Costa Rican sequences for the species *Ptenos leucopoda*. It is possible, however, that the monophyly of the Argidae group of 39 MOTUs could be an artifact resulting from undersampling of alternative host plant groups in the Neotropics. Nevertheless, the fact that related sawflies have not been found on other hosts in Guanacaste, Costa Rica ^6^ despite many years of sampling, suggests that this sawfly clade is genuinely restricted to *Inga* and close relatives.

The genus *Inga* is thought to represent a geologically young radiation, with a common ancestor between 4 and 10 million years ago (Richardson et al., 2001). If associated sawflies have co-diversified with their *Inga* hosts, we expect the ages of the two radiations to be similar. Because there are no fossil records for the *Inga*-associated Argidae clade, we used independent estimates for beetles and butterflies in order to calibrate the *Inga*-associated sawfly phylogeny. Comparisons with fossil-calibrated phylogenies for other sawfly taxa suggests that these calibrations are broadly applicable to sawflies (Nyman et al., 2006; Malm and Nyman, 2015). Based on these data, the estimate we obtained for the age of the common ancestor of the *Inga-*associated Argidae clade suggests that this group diversified at broadly the same time, or more recently, than their plant hosts [mean of 6.27 (between 4.78 and 7.93) million years ago using the Brower (1994) estimate and a mean of 5.31 (between 4.05 and 6.72) million years ago using the Papadopoulou et al. (2010) estimate]. Given the uncertainty in the date of the *Inga* radiation, these results are consistent with *Inga*-associated sawflies having diversified alongside their hosts, a conservative pattern of host plant use also found in other sawfly clades (Nyman et al., 2010; Schmidt and Walter, 2014) and in leaf-feeding beetles, seed predators and many other insect herbivore groups (Farrel and Mitter, 1998; Janz and Nylin, 1998; Winkler and Mitter, 2008; Edger et al., 2015). Alternatively, the radiation of Argidae might be younger than *Inga*, a pattern consistent with host-resource tracking or ecological fitting.

Ehrlich and Raven (1964) hypothesized that any taxonomic correspondence between plants and herbivores was the result of herbivore tracking of phylogenetically conserved host plant traits. Several lines of evidence suggest that defensive chemistry plays the key role in structuring sawfly associations with *Inga*. First, among all host traits, chemistry was identified as the most important predictor in sawfly *Inga* associations, with sawflies preferring *Inga* hosts that express amines (**Figure 2**). Second, after controlling for phylogenetic effects, we find that host associations in sawflies are more strongly correlated with *Inga* chemistry than *Inga* phylogeny (**Table 2** and **Figures 3A,B**). The significant concordance between the topologies of *Inga* and sawfly phylogenies could thus be explained as the result of phylogenetic conservatism in *Inga* chemistry for the set of species attacked by sawflies. Chemistry is better able to explain *Inga-*sawfly associations than the *Inga* phylogeny alone because some sawfly sister taxa are associated with hosts that are chemically similar but not closely related (**Figures 3A,B**), while there are very few cases of sawfly sister MOTUs associated with chemically divergent hosts.

Phylogenetic concordance between plants and herbivores could represent either a signature of codiversification or a radiation onto existing *Inga* (delayed resource tracking). The facts that host-shifting in sawflies is more strongly determined by *Inga* defenses than by *Inga* phylogeny (**Table 2** and **Figures 3A,B**), and that most examples of shifts between *Inga* hosts include species that are similar in defensive chemistry, regardless of relatedness (**Figures 3A,B**), support delayed host tracking. Nevertheless, it is striking that none of the more basal species in the *Inga* phylogeny are attacked by sawflies (**Figure 4**). This strongly implies cospeciation, that the ancestors of both the argid and tenthredinid sawflies now associated with *Inga* colonized, and then codiversified alongside an already ongoing radiation of *Inga*. In the end, which hypothesis is correct depends on the relative ages of the *Inga* and sawfly radiations. Our best estimate of the age of the common ancestor of the *Inga*-associated Argidae is fairly constrained (4.02–7.93 million years). In contrast, our estimate for the age of the common ancestor of *Inga* ranges from 4 to 10 million years, with the further caveat that the more derived *Inga* that are sawfly hosts are younger by an unknown extent. While the dates used here are consistent with codiversification, delayed resource tracking cannot be ruled out until the dates of origin for both crown groups, particularly *Inga*, are known with more certainty.

Although the significance of defensive traits in plant-herbivore diversification has been recognized (Futuyma and Agrawal, 2009), it is often not included in coevolutionary studies. Most studies compare the congruence between the ages and topologies of insect and host-plant phylogenies with the expectation that closely related hosts will share closely related herbivores (reviewed in Suchan and Alvarez, 2015). Alternative hypotheses, such as tracking of host defenses, cannot be tested. We argue that in order to understand the process and factors that influence the evolution of herbivore host ranges, characterization of relevant host traits is essential.

### *Inga*-Sawfly Patterns of Diversification

Previous work suggests that modes of speciation vary among sawfly lineages with different life history strategies. Analyses of temperate nematine sawflies suggest that lineages with externally feeding larvae tend to feed on multiple host plant species (Nyman et al., 2006, 2010), and, as a result are more likely to diversify through allopatric speciation than via host shifts. In contrast, gall-inducing sawfly lineages, which are more intimately associated metabolically with their hosts, are both more likely to feed on a narrow host range and to diversify by shifts among willow host species (Nyman et al., 2006).

Although they are external feeders, the narrow host ranges observed for *Inga*- and *Zygia-* feeding sawflies (1-2 hosts per MOTU) more closely match patterns seen in specialist gall-inducing sawflies than the wider host associations seen in externally feeding sawflies on willow. This high host specificity could result from constraints or adaptations related to host use, such as host-finding capabilities, avoidance of larval predators, and avoidance or sequestration of host toxins (Brooks and McLennan, 2002). For the sawflies associated with *Inga*, a control choice experiment in a previous study suggested that host preference is primarily driven by leaf secondary metabolites and possibly nutrition (Endara et al., 2015). Although much of the available literature concerns the superfamily Tenthredinidae in the northern hemisphere, and the families Pergidae and Argidae in Australia, many sawflies show adaptations for dealing with, and using the host plant chemistry. Many can sequester and modify toxic host compounds for use in their own anti-predator defense [e.g., Diprionidae (Eisner et al., 1974); Tenthredinidae (Boevé et al., 2013); Argidae (Petre et al., 2007)], particularly against ants (Boevé and Schaffner, 2003; Petre et al., 2007; Boevé et al., 2013). This is particularly relevant in *Inga*, many species of which recruit ant guards through secretion of extrafloral nectar. The lack of any significant association between the presence of ants and sawflies on *Inga* suggests that sawflies may not be highly sensitive to ants that provide some defense against other herbivores (Endara et al., 2017). In *Inga*, we observed that when contacted by ants, sawfly larvae raised their abdomen, and ants generally retreated immediately (MJ Endara, personal observation). In addition, most of the sawfly MOTUs found on *Inga* are gregarious, a characteristic often considered a sign of chemical defense (Boevé et al., 2013). Thus, sawflies associated with *Inga* may have an intimate relationship with their host chemistry.

Although the specialized relationship between sawflies and *Inga* would suggest a mode of speciation similar to the specialist, gall-inducing sawflies, our phylogenetic analysis reveals that the predominant mode of speciation is allopatric, similar to external sawfly feeders on willow (Nyman et al., 2010). Results from the evolutionary analysis that included phylogenetic and chemical effects show that the coevolutionary effect best explained variation in sawfly incidence when between-region information was included (**Table 2**). This suggests that pairs of sister *Inga* host populations and sawfly MOTUs occur in non-overlapping geographic regions (Hadfield et al., 2014). This pattern can be seen throughout the whole sawfly phylogeny, with more than 60% of lineage splits potentially caused by non-ecological factors in allopatry. For example, MOTU 31 attacks *Inga alba* in Peru, and its sister species MOTU 32 is associated with *Inga alba* in French Guiana (**Figures 3A,B**). This is evidence for allopatric speciation between sawfly sister taxa associated with the same *Inga* host (Barraclough and Vogler, 2000). Thus, *Inga*-feeding sawflies could have diverged and speciated in allopatry either directly because of *Inga* speciation or because the same ecological and geographical factors that facilitated *Inga* speciation could have facilitated the speciation of its sawfly herbivores. Alternatively, although species accumulation curves show that further sampling would not yield many additional *Inga*-sawfly interactions, we may have missed collecting sister sawfly species at the same site, meaning that speciation in sympatry cannot be totally ruled out.

The finding that the speciation process in the *Inga*-sawflies is largely non-ecological in allopatry does not exclude the possibility that some diversification events may have an ecological basis (i.e., host shifts). Along the phylogeny, four instances of lineage splits can potentially be ecologically based, with two host shifts to novel hosts in sympatry (MOTU 36 is associated with *Inga ruiziana* in Ecuador which produces phenolics, whereas the sister species MOTU 37 is associated with *Inga auristellae* which produces saponins, **Figures 3A,B**) and in allopatry (MOTU 7 is associated with *Inga marginata* in French Guiana which produces saponins, and the sister lineage MOTU 8 attacks *Inga umbellifera* in Panama which produces amines, **Figures 3A,B**). The other two host shifts simply involved range expansion (i.e., switch to a different host but with a similar chemistry), with one example in sympatry (in French Guiana, MOTU 7 is associated with *Inga obidensis* and MOTU 8 is attacking *Inga jenmanii*, both hosts produce amines, **Figures 3A,B**) and the other in allopatry (MOTU 16 is associated with *Inga edulis* in Ecuador and MOTU 18 is associated with *Inga thibaudiana* in French Guiana, with both hosts producing phenolics, **Figures 3A,B**). Excluding few exceptions, none of these switches involved phylogenetically closely related hosts, but rather chemically similar ones (**Figures 3A,B**), highlighting the importance of plant chemistry in ecological speciation.

## Conclusion

Our phylogeny- and trait-based analysis of the interactions between *Inga* and Argidae sawflies indicates the importance of including ecologically relevant traits for host selection in studies of herbivore-host plant coevolution. For example, closely related sawfly species often shift to *Inga* that are similar chemically but not closely related phylogenetically. Our results suggest a major role for host chemistry in explaining both the observed concordance between *Inga* and sawfly phylogenies, and in explaining the deviations from this pattern resulting from evolutionary tracking of defensive traits by sawflies.

Our analyses suggest two modes of diversification of sawflies: (i) allopatric divergence between sawfly sister taxa associated with the same *Inga* food plant and (ii) niche shifts. The vast majority of lineage splits in these sawflies seem to have occurred non-ecologically in allopatry, a pattern that may well be true for other groups of insect herbivores (Nyman et al., 2010). Thus, sawflies primarily speciate allopatrically, but descendent species are constrained to use the same host species or others with similar chemistry. Closely related sawflies very rarely attack chemically dissimilar *Inga* species, implying that, for the most part, these herbivores have not experienced the niche shifts thought to promote diversification in other insect herbivores, and particularly in highly specialized taxa (Rundle and Nosil, 2005; Dyer et al., 2007; Futuyma and Agrawal, 2009).

## Author Contributions

M-JE, JN, PC, GS, and TK designed and conducted the research. M-JE, KD, and GS designed and performed the data analysis. DF and GY contributed to the metabolomic analysis. JN, RP, KD, CK, and GS contributed the next-generation DNA sequence data and phylogenies. M-JE, JN, PC, KD, DF, GY, RP, CK, GS, and TK wrote the manuscript.

## Conflict of Interest Statement

The authors declare that the research was conducted in the absence of any commercial or financial relationships that could be construed as a potential conflict of interest.

## Footnotes

1 https://gnps.ucsd.edu/ProteoSAFe/static/gnps-splash.jsp

2 https://github.com/ColeyKursarLab/endara_sawflies_2018

3 http://boldsystems.org

4 doi: 10.5061/dryad.8403km4

5 https://gnps.ucsd.edu/ProteoSAFe/static/gnps-splash.jsp

6 janzen.sas.upenn.edu/index.html

## Supplementary Material

Sawfly sequence data can be found in GenBank, accessions MH206848 – MH207017 for COI, MH206768 – MH206847 for ITS2, MH206617 – MH206691 for *wg* and MH206692 – MH206767 for *PGD*.

FIGURE S1 MrBayes majority-rule consensus tree for the mitochondrial *COI* DNA barcode fragment. Numbers above nodes indicate posterior probabilities. Taxon label colors indicate membership of 1.5% sequence divergence jMOTU taxa, indicated by the labels at right.

FIGURE S2 Results of MOTU identification analyses of *Inga-* and *Zygia-*feeding sawflies, using a 645 bp fragment of the mitochondrial *COI* DNA barcoding region for **(a)** jMOTU and **(b)** ABGD.

FIGURE S3 MrBayes majority-rule consensus tree for the nuclear locus *ITS2*, sequenced for exemplars of each of the selected 41 jMOTU 1.5% *COI* MOTUs. Numbers above nodes indicate posterior probabilities. Taxon labels are colored to indicate membership of different MOTUs.

FIGURE S4 MrBayes majority-rule consensus tree for the nuclear locus *wingless*, sequenced for exemplars of each of the selected 41 jMOTU 1.5% *COI* MOTUs. Numbers above nodes indicate posterior probabilities. Taxon labels are colored to indicate membership of different MOTUs.

FIGURE S5 Sawfly MOTU accumulation curves when sampling over *Inga* host plant taxa, and when sampling over individuals. For each curve, the mean estimate is shown as a dark blue line and the standard deviation as a pale blue shaded region either side. The total numbers of *Inga* taxa and sawfly specimens in these analyses were 34 and 1286, respectively.

FIGURE S6 Phylogenetic relationships for the gene *CO1* among the *Inga*-feeding sawfly MOTUs and a panel of voucher sequences for sawflies in the families Argidae, Pergidae (sister group to Argidae; Malm and Nyman, 2015) and Tenthredinidae. The tree shown is a majority-rule consensus tree constructed in MrBayes, using substitutions modeled as GTR+I+G for each of 1st and 2nd codon positions, and GTR+G for 3rd positions. We used a relaxed clock, with a birth-death speciation model. Numbers at nodes indicate posterior probability. Taxon labels are colored by sampling source: red MOTU numbers are larvae found feeding on *Inga* or *Zygia*, while other colors indicate reference sequences for adult Argidae, Pergidae and Tenthredinidae. The taxon label MOTU17_BCI marked with two asterisks is a voucher sequence for a specimen of *Ptenos leucoopoda* (Argidae) sampled from *Inga oerstediana* (and also recorded from *I. vera*) in Costa Rica (Smith et al., 2013).

TABLE S1 List of compounds putatively identified through matches to reference MSMS spectra on the Global Natural Products Social Molecular Networking database (https://gnps.ucsd.edu/ProteoSAFe/static/gnps-splash.jsp). The cosine score is a measure of the similarity of MS/MS-derived fragments between two compounds.

TABLE S2 Metadata for all sawfly specimens collected in this study, including host plant and collection location, MOTU allocation (1.5% jMOTU taxa), and Genbank accession numbers for all sequenced gene fragments. Note that in our sampling system, each study site has independent collection numbers. Thus, it is possible for two *Inga* plants to have the same host plant number, but only because they were sampled at different sites.

TABLE S3 Metadata for additional reference sawfly sequences, with species name, country of origin, Genbank accession numbers for *COI* and *PGD* gene fragments, and source reference.

TABLE S4 Information on the ten sequence loci used for construction of the *Inga* species tree. Locus number, reference transcript, functional annotation and the substitution model used in phylogenetic analyses all refer to Nicholls et al. (2015).

TABLE S5 *Parafit* analysis output for sawfly and *Inga* phylogenies, for sawfly MOTUs in the family Argidae. **(B)** *Parafit* analysis of concordance between sawfly phylogeny and Inga chemogram. In **(A)** and **(B)** herbivore-*Inga* associations that are identified as individually significant are highlighted in yellow.

APPENDIX SI Molecular methods for PCR amplification of sawfly sequences.

APPENDIX SII Detailed chemical methods for construction of a chemical similarity matrix.
