# Supplementary material for: Tracking of Host Defenses and Phylogeny During the Radiation of Neotropical Inga-Feeding Sawflies (Hymenoptera; Argidae)
Source: Front Plant Sci. 2018 Aug 23;9:1237. doi: 10.3389/fpls.2018.01237 (PMC6116116; doi:10.3389/fpls.2018.01237)
Supplement: FIGURE S1 — MrBayes majority-rule consensus tree for the mitochondrial COI DNA barcode fragment. Numbers above nodes indicate posterior probabilities. Taxon label colors indicate membership of 1.5% sequence divergence jMOTU taxa, indicated by the labels at right. [file Image_1.PDF]

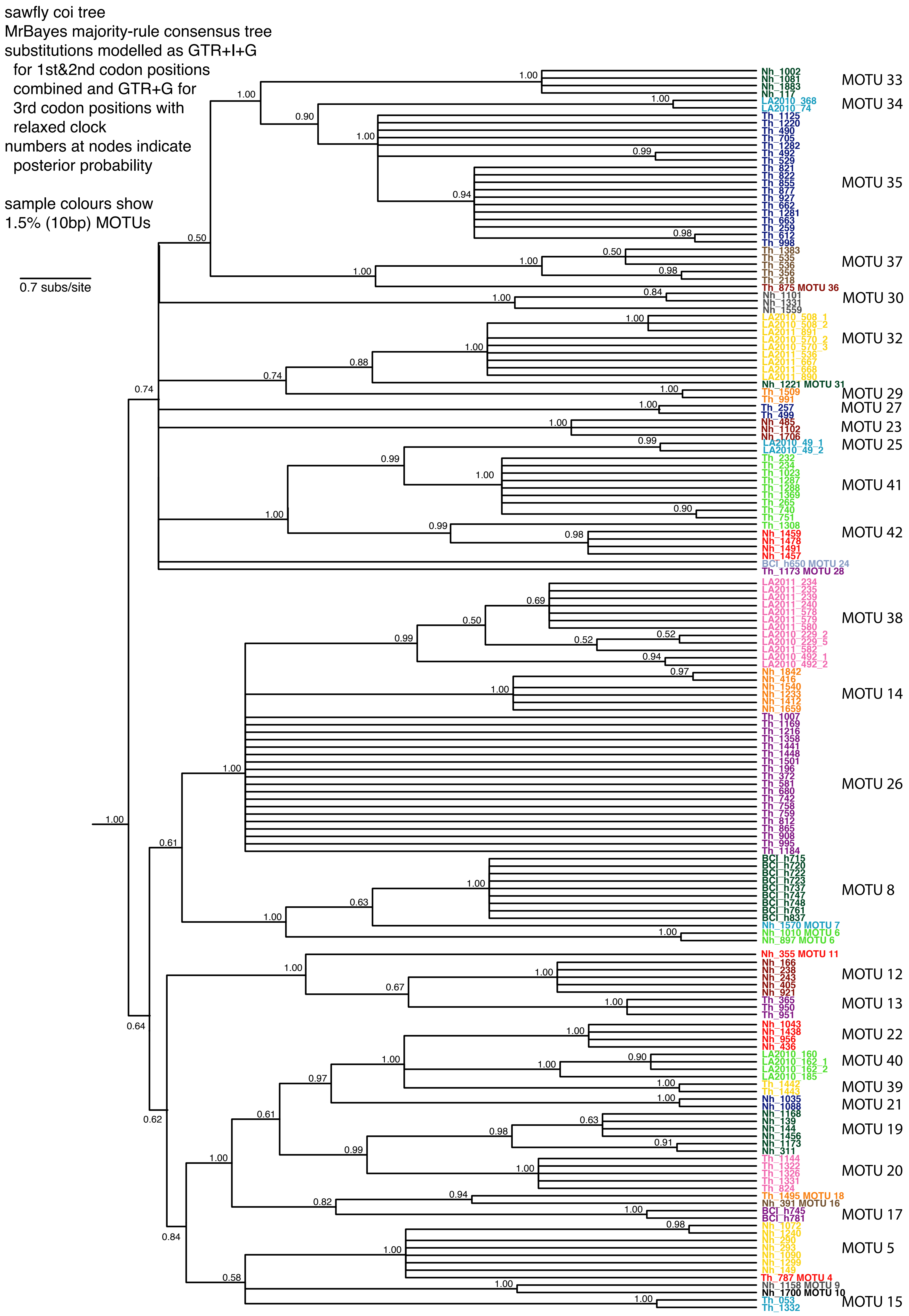
sawfly coi tree
MrBayes majority-rule consensus tree
substitutions modelled as GTR+I+G
for 1st&2nd codon positions
combined and GTR+G for
3rd codon positions with
relaxed clock
numbers at nodes indicate
posterior probability
sample colours show
1.5% (10bp) MOTUs
0.7 subs/site
1.00
1.00
0.90
1.00
1.00
0.99
0.94
0.98
0.50
1.00
0.50
1.00
0.98
0.84
1.00
1.00
1.00
0.88
0.74
0.74
1.00
1.00
1.00
0.99
0.99
1.00
0.90
1.00
0.99
0.98
0.69
0.50
0.99
0.52
0.52
0.94
0.97
1.00
1.00
1.00
0.61
1.00
0.63
1.00
1.00
1.00
1.00
0.67
1.00
0.64
1.00
1.00
0.90
1.00
0.97
1.00
1.00
0.61
0.63
0.98
0.91
0.62
0.99
1.00
1.00
0.94
0.82
1.00
0.98
0.84
1.00
0.58
1.00
1.00
Nh_1002
Nh_1081
Nh_1883
Nh_117
MOTU 33
LA2010_368
LA2010_74
MOTU 34
Th_1125
Th_1220
Th_490
Th_705
Th_1282
Th_492
Th_529
Th_821
Th_822
Th_855
Th_877
Th_927
Th_662
Th_1281
Th_663
Th_259
Th_612
Th_998
MOTU 35
Th_1383
Th_535
Th_536
Th_356
Th_218
MOTU 37
Th_875 MOTU 36
Nh_1101
Nh_1331
Nh_1559
MOTU 30
LA2010_508_1
LA2010_508_2
LA2011_891
LA2010_570_2
LA2010_570_3
LA2011_536
LA2011_667
LA2011_668
LA2011_890
MOTU 32
Nh_1221 MOTU 31
Th_1509
Th_991
MOTU 29
Th_257
Th_499
MOTU 27
Nh_485
Nh_1102
Nh_1706
MOTU 23
LA2010_49_1
LA2010_49_2
MOTU 25
Th_232
Th_234
Th_1023
Th_1287
Th_1288
Th_1369
Th_265
Th_740
Th_751
MOTU 41
Th_1308
Nh_1459
Nh_1478
Nh_1491
Nh_1457
MOTU 42
BCI_h650 MOTU 24
Th_1173 MOTU 28
LA2011_234
LA2011_235
LA2011_239
LA2011_240
LA2011_578
LA2011_579
LA2011_580
LA2010_229_2
LA2010_229_5
LA2011_582
LA2010_492_1
LA2010_492_2
MOTU 38
Nh_1842
Nh_416
Nh_1540
Nh_1233
Nh_1412
Nh_1659
MOTU 14
Th_1007
Th_1169
Th_1216
Th_1358
Th_1441
Th_1448
Th_1501
Th_196
Th_372
Th_581
Th_680
Th_742
Th_758
Th_759
Th_812
Th_865
Th_908
Th_995
Th_1184
MOTU 26
BCI_h715
BCI_h720
BCI_h722
BCI_h723
BCI_h737
BCI_h747
BCI_h748
BCI_h761
BCI_h837
MOTU 8
Nh_1570 MOTU 7
Nh_1010 MOTU 6
Nh_897 MOTU 6
Nh_355 MOTU 11
Nh_166
Nh_238
Nh_243
Nh_405
Nh_921
MOTU 12
Th_365
Th_950
Th_951
MOTU 13
Nh_1043
Nh_1438
Nh_956
Nh_436
MOTU 22
LA2010_160
LA2010_162_1
LA2010_162_2
LA2010_185
MOTU 40
Th_1442
Th_1443
MOTU 39
Nh_1035
Nh_1088
MOTU 21
Nh_1168
Nh_139
Nh_144
Nh_1456
Nh_1173
Nh_311
MOTU 19
Th_1144
Th_1322
Th_1326
Th_1331
Th_824
MOTU 20
Th_1495 MOTU 18
Nh_391 MOTU 16
BCI_h745
BCI_h781
MOTU 17
Nh_1072
Nh_1240
Nh_290
Nh_293
Nh_1090
Nh_1299
Nh_149
MOTU 5
Th_787 MOTU 4
Nh_1158 MOTU 9
Nh_1700 MOTU 10
Th_053
Th_1332
MOTU 15
