# Supplementary figures and images for: Tracking of Host Defenses and Phylogeny During the Radiation of Neotropical Inga-Feeding Sawflies (Hymenoptera; Argidae)
Source: Front Plant Sci. 2018 Aug 23;9:1237. doi: 10.3389/fpls.2018.01237 (PMC6116116; doi:10.3389/fpls.2018.01237)

Figure S2

(a) JMOTU output

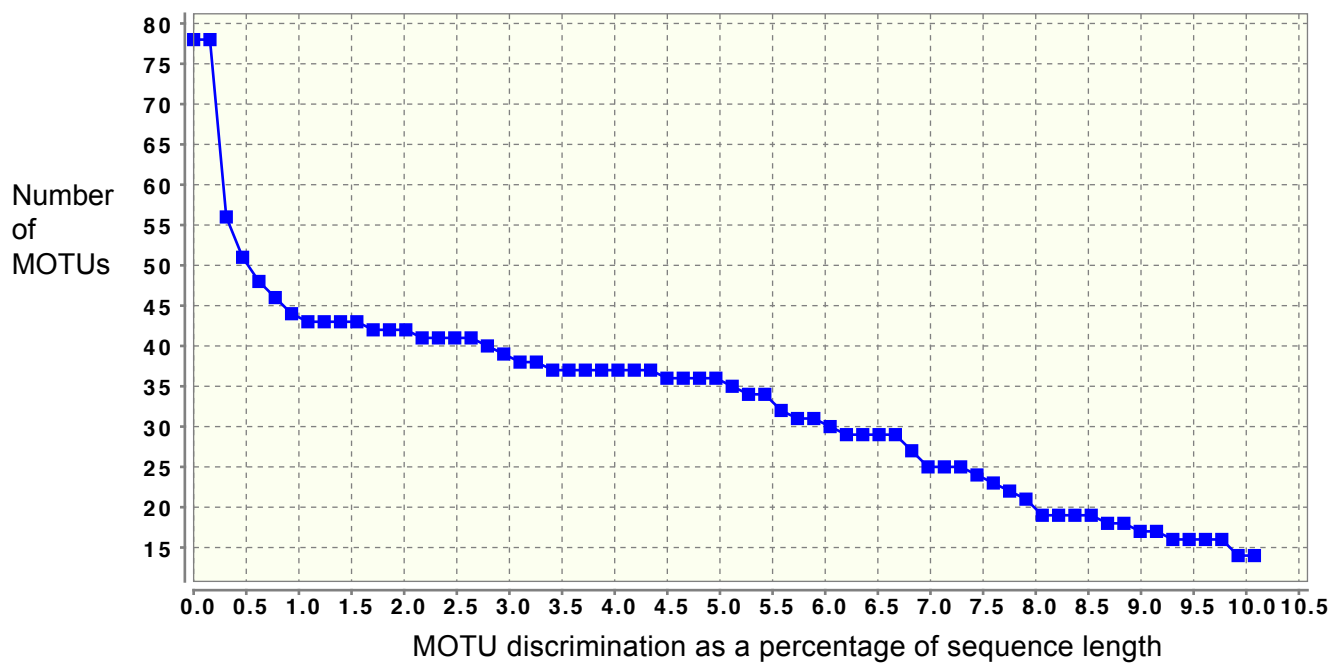

(b) ABGD output

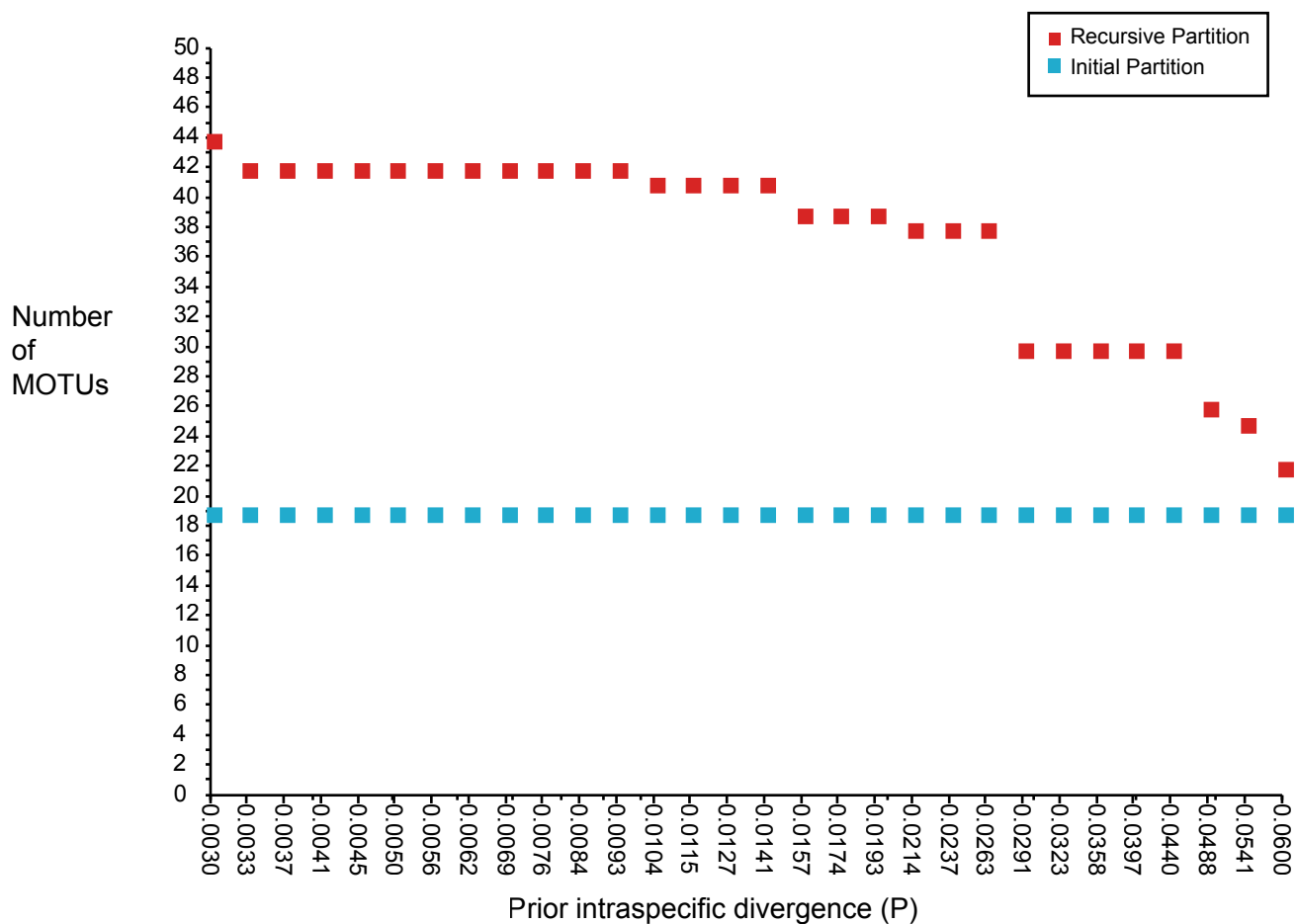

Figure S2

(a) JMOTU output

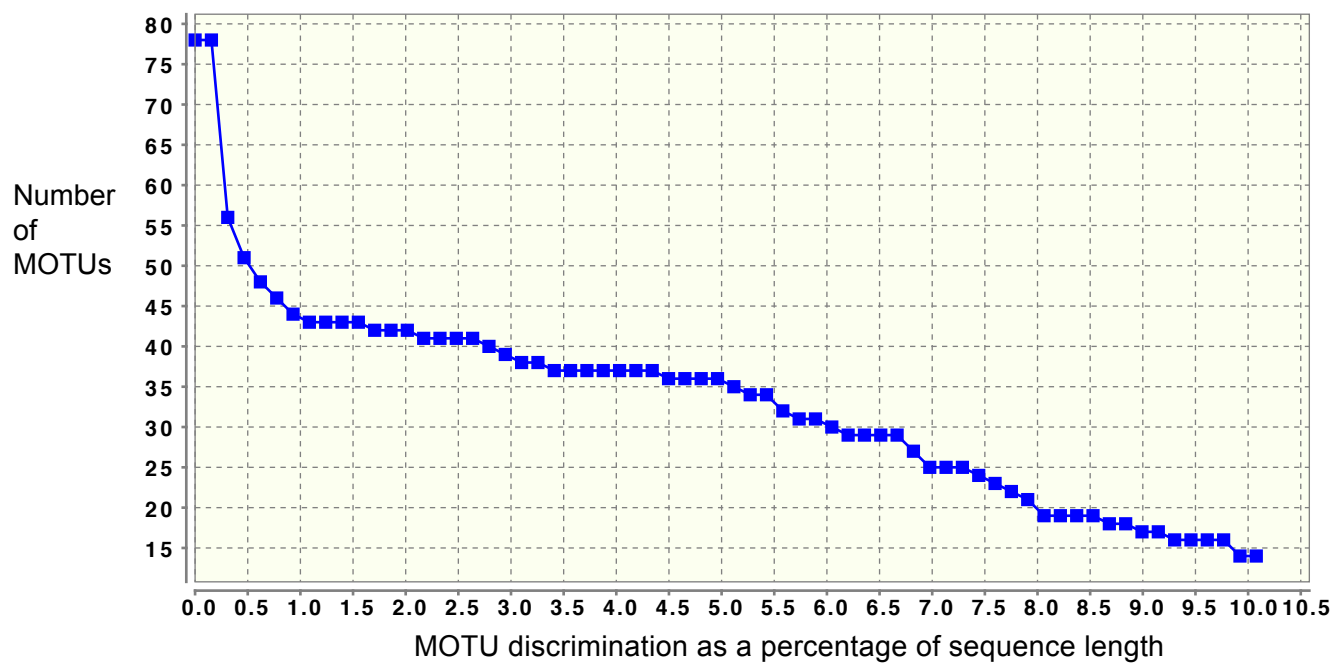

(b) ABGD output

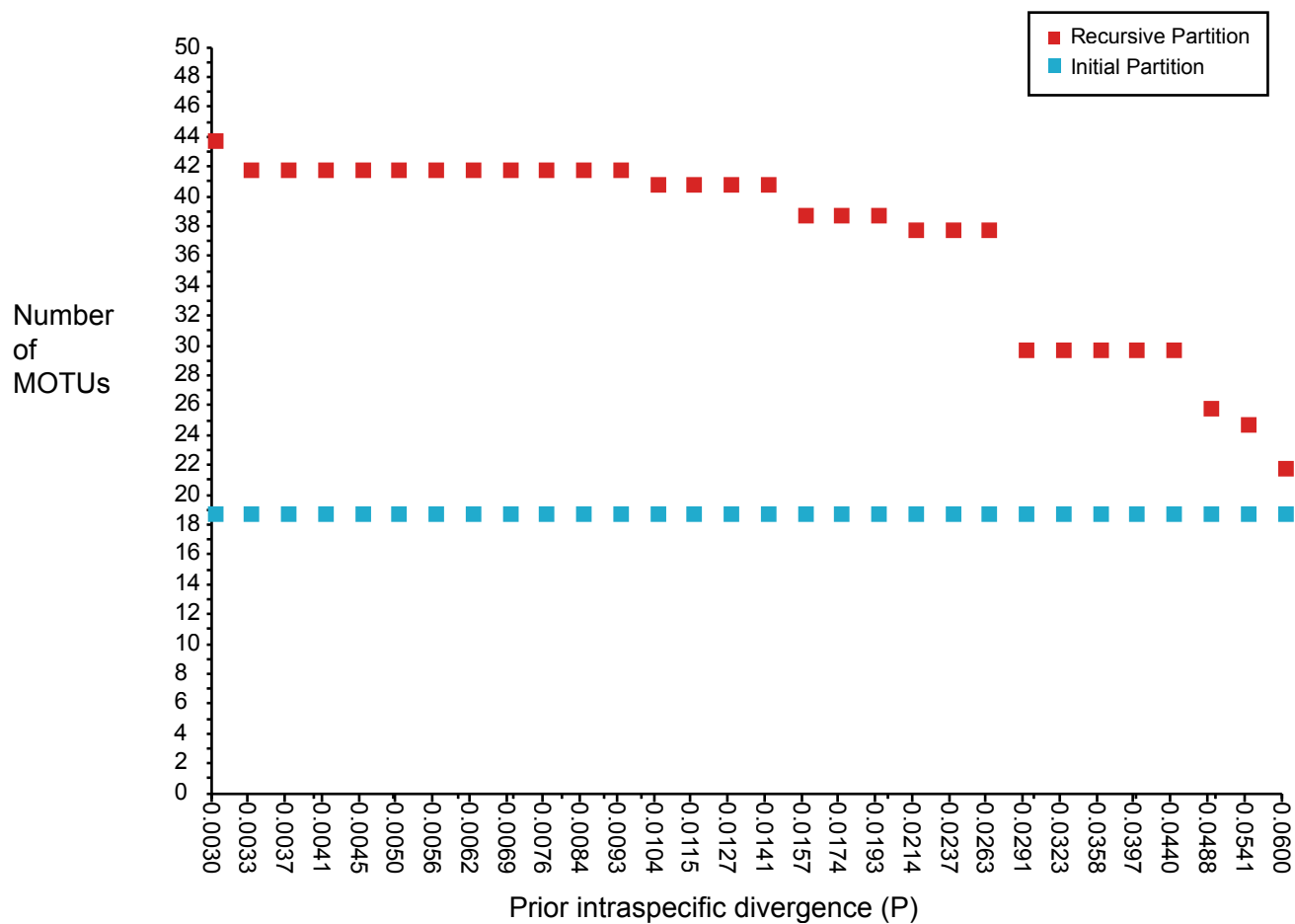

Supplement: FIGURE S2 — Results of MOTU identification analyses of Inga- and Zygia-feeding sawflies, using a 645 bp fragment of the mitochondrial COI DNA barcoding region for (a) jMOTU and (b) ABGD. [file Image_2.PDF]

Sample colours and numbers  
show CO1 1.5% (10bp) MOTUs

0.0060 subs/site

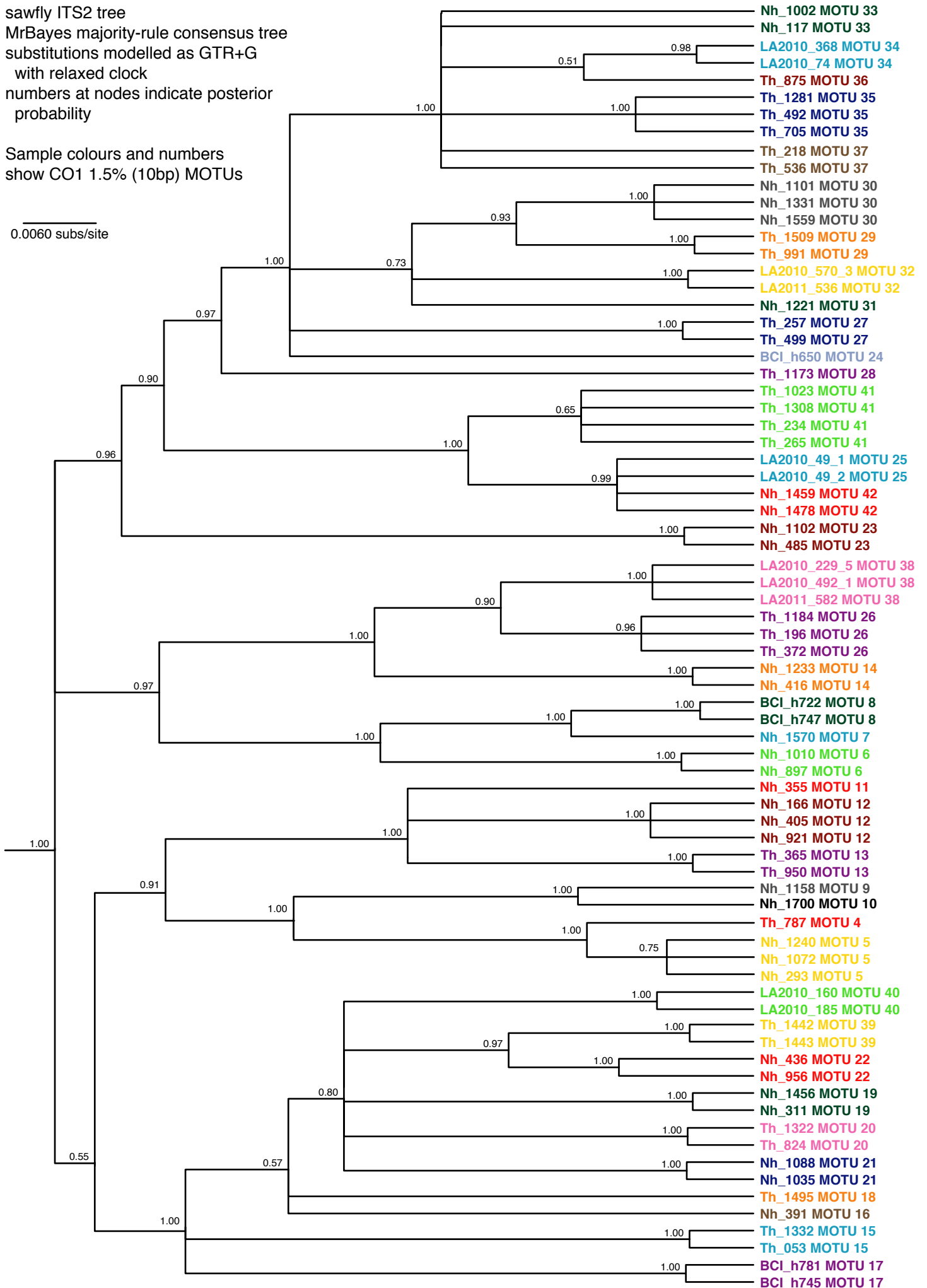

Supplement: FIGURE S3 — MrBayes majority-rule consensus tree for the nuclear locus ITS2, sequenced for exemplars of each of the selected 41 jMOTU 1.5% COI MOTUs. Numbers above nodes indicate posterior probabilities. Taxon labels are colored to indicate membership of different MOTUs. [file Image_3.PDF]

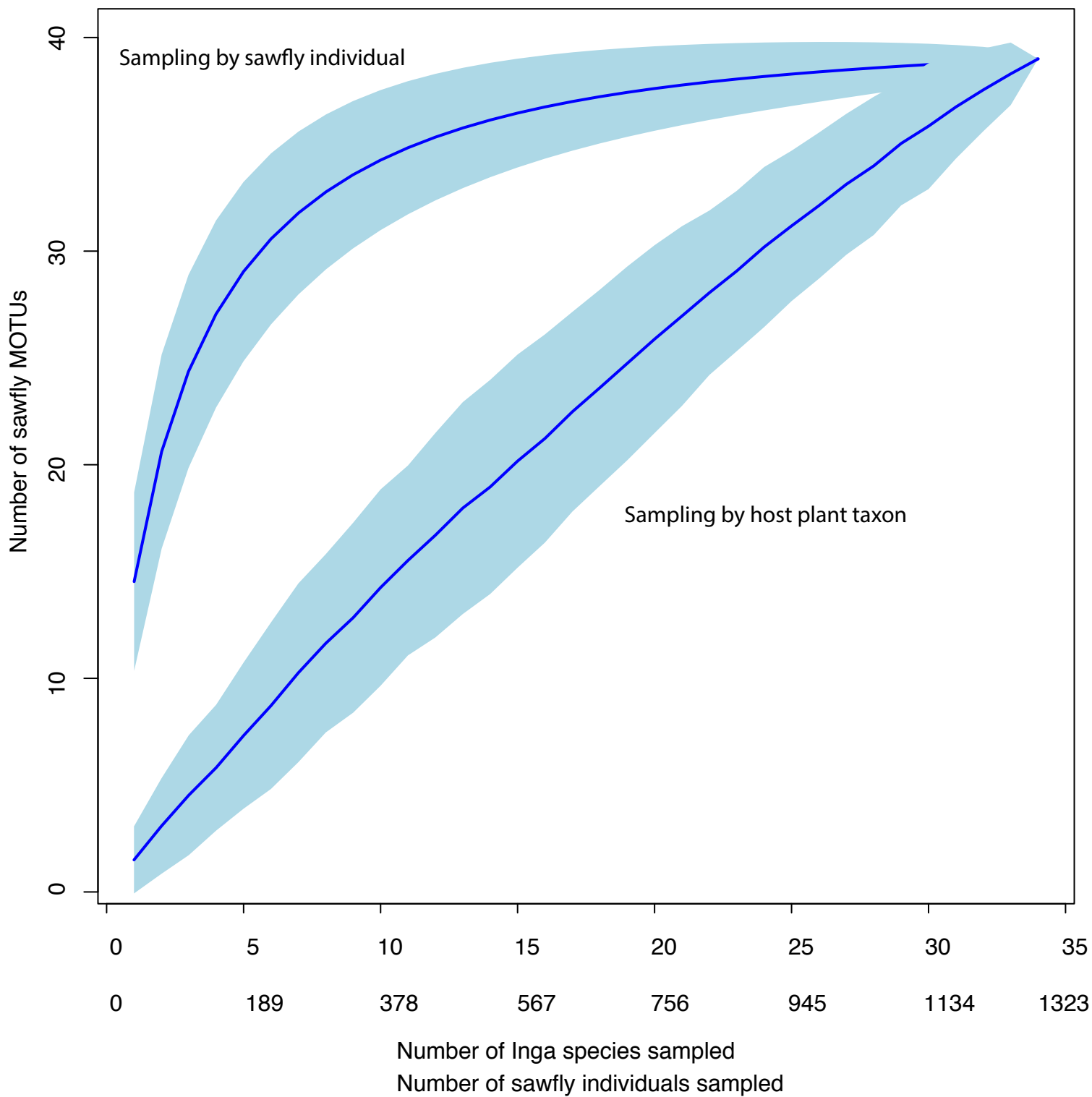

Supplement: FIGURE S5 — Sawfly MOTU accumulation curves when sampling over Inga host plant taxa, and when sampling over individuals. For each curve, the mean estimate is shown as a dark blue line and the standard deviation as a pale blue shaded region either side. The total numbers of Inga taxa and sawfly specimens in these analyses were 34 and 1286, respectively. [file Image_5.PDF]

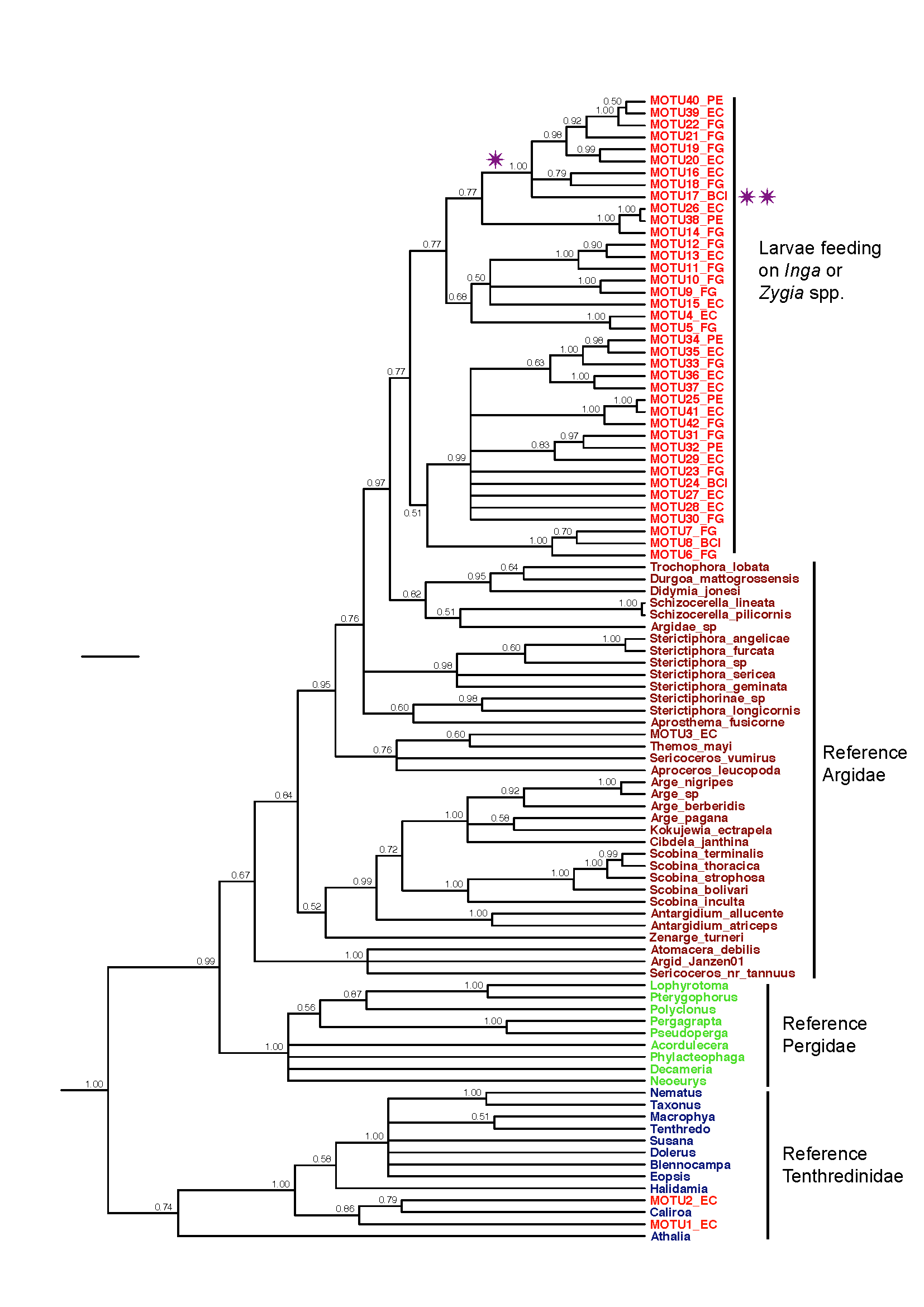

Supplement: FIGURE S6 — Phylogenetic relationships for the gene CO1 among the Inga-feeding sawfly MOTUs and a panel of voucher sequences for sawflies in the families Argidae, Pergidae (sister group to Argidae; Malm and Nyman, 2015) and Tenthredinidae. The tree shown is a majority-rule consensus tree constructed in MrBayes, using substitutions modeled as GTR+I+G for each of 1st and 2nd codon positions, and GTR+G for 3rd positions. We used a relaxed clock, with a birth-death speciation model. Numbers at nodes indicate posterior probability. Taxon labels are colored by sampling source: red MOTU numbers are larvae found feeding on Inga or Zygia, while other colors indicate reference sequences for adult Argidae, Pergidae and Tenthredinidae. The taxon label MOTU17_BCI marked with two asterisks is a voucher sequence for a specimen of Ptenos leucoopoda (Argidae) sampled from Inga oerstediana (and also recorded from I. vera) in Costa Rica (Smith et al., 2013). [file Image_6.TIF]

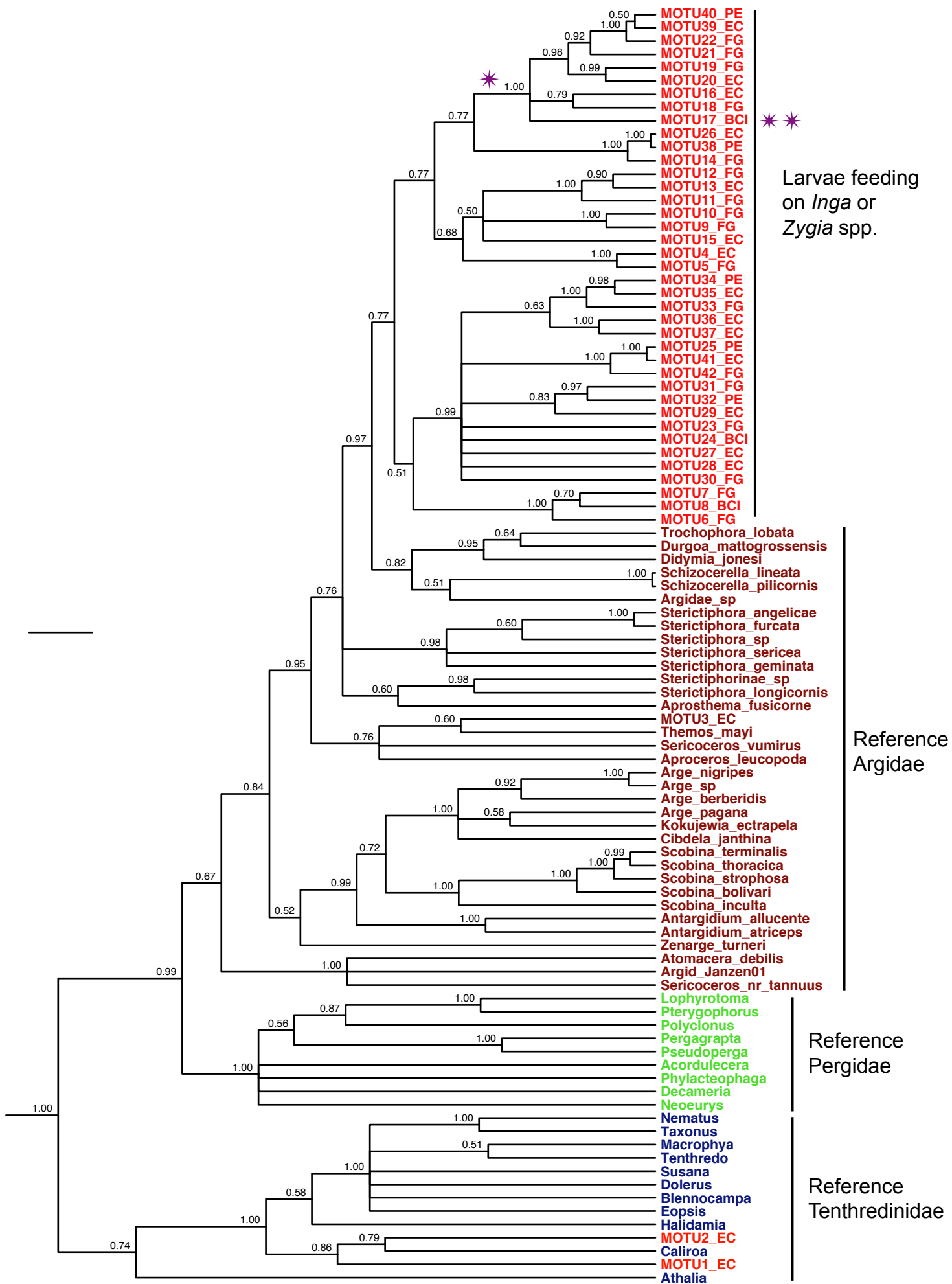

Supplement: APPENDIX SII — Detailed chemical methods for construction of a chemical similarity matrix. [file Data_Sheet_2.PDF]
