# Supplementary material for: Tracking of Host Defenses and Phylogeny During the Radiation of Neotropical Inga-Feeding Sawflies (Hymenoptera; Argidae)
Source: Front Plant Sci. 2018 Aug 23;9:1237. doi: 10.3389/fpls.2018.01237 (PMC6116116; doi:10.3389/fpls.2018.01237)
Supplement: FIGURE S4 — MrBayes majority-rule consensus tree for the nuclear locus wingless, sequenced for exemplars of each of the selected 41 jMOTU 1.5% COI MOTUs. Numbers above nodes indicate posterior probabilities. Taxon labels are colored to indicate membership of different MOTUs. [file Image_4.PDF]

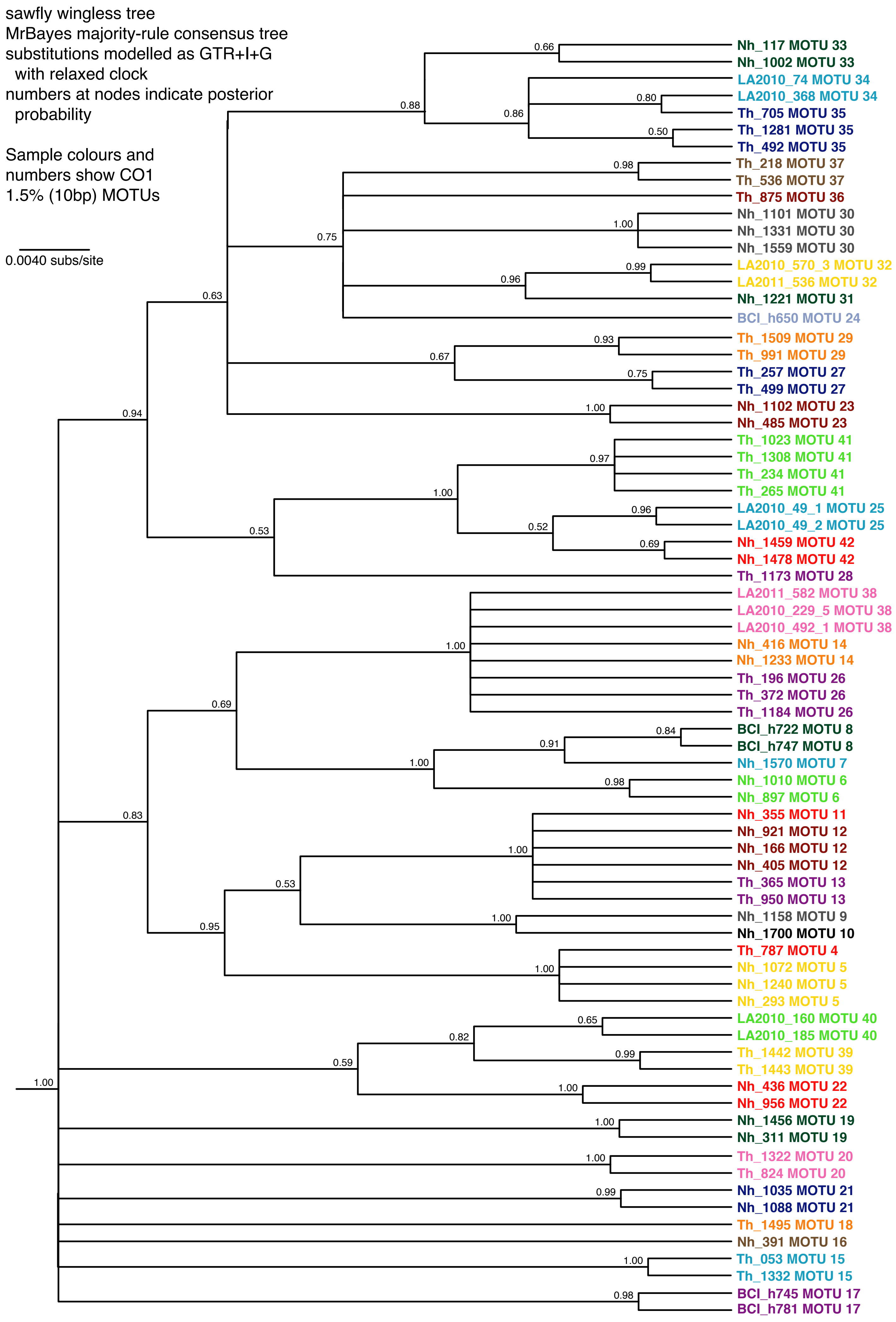
sawfly wingless tree
MrBayes majority-rule consensus tree
substitutions modelled as GTR+I+G
with relaxed clock
numbers at nodes indicate posterior
probability
Sample colours and
numbers show CO1
1.5% (10bp) MOTUs
0.0040 subs/site
Nh_117 MOTU 33
Nh_1002 MOTU 33
LA2010_74 MOTU 34
LA2010_368 MOTU 34
Th_705 MOTU 35
Th_1281 MOTU 35
Th_492 MOTU 35
Th_218 MOTU 37
Th_536 MOTU 37
Th_875 MOTU 36
Nh_1101 MOTU 30
Nh_1331 MOTU 30
Nh_1559 MOTU 30
LA2010_570_3 MOTU 32
LA2011_536 MOTU 32
Nh_1221 MOTU 31
BCI_h650 MOTU 24
Th_1509 MOTU 29
Th_991 MOTU 29
Th_257 MOTU 27
Th_499 MOTU 27
Nh_1102 MOTU 23
Nh_485 MOTU 23
Th_1023 MOTU 41
Th_1308 MOTU 41
Th_234 MOTU 41
Th_265 MOTU 41
LA2010_49_1 MOTU 25
LA2010_49_2 MOTU 25
Nh_1459 MOTU 42
Nh_1478 MOTU 42
Th_1173 MOTU 28
LA2011_582 MOTU 38
LA2010_229_5 MOTU 38
LA2010_492_1 MOTU 38
Nh_416 MOTU 14
Nh_1233 MOTU 14
Th_196 MOTU 26
Th_372 MOTU 26
Th_1184 MOTU 26
BCI_h722 MOTU 8
BCI_h747 MOTU 8
Nh_1570 MOTU 7
Nh_1010 MOTU 6
Nh_897 MOTU 6
Nh_355 MOTU 11
Nh_921 MOTU 12
Nh_166 MOTU 12
Nh_405 MOTU 12
Th_365 MOTU 13
Th_950 MOTU 13
Nh_1158 MOTU 9
Nh_1700 MOTU 10
Th_787 MOTU 4
Nh_1072 MOTU 5
Nh_1240 MOTU 5
Nh_293 MOTU 5
LA2010_160 MOTU 40
LA2010_185 MOTU 40
Th_1442 MOTU 39
Th_1443 MOTU 39
Nh_436 MOTU 22
Nh_956 MOTU 22
Nh_1456 MOTU 19
Nh_311 MOTU 19
Th_1322 MOTU 20
Th_824 MOTU 20
Nh_1035 MOTU 21
Nh_1088 MOTU 21
Th_1495 MOTU 18
Nh_391 MOTU 16
Th_053 MOTU 15
Th_1332 MOTU 15
BCI_h745 MOTU 17
BCI_h781 MOTU 17
