# Supplementary material for: Tracking of Host Defenses and Phylogeny During the Radiation of Neotropical Inga-Feeding Sawflies (Hymenoptera; Argidae)
Source: Front Plant Sci. 2018 Aug 23;9:1237. doi: 10.3389/fpls.2018.01237 (PMC6116116; doi:10.3389/fpls.2018.01237)
Supplement: APPENDIX SI — Molecular methods for PCR amplification of sawfly sequences. [file Data_Sheet_1.doc]

**Supplementary Information**

**Appendix SI:** Molecular methods for PCR amplification of sawfly sequences.

**1. Cytochrome *c* oxidase subunit I (COI)**

Primers used amplify the standard COI barcoding region, using either LCO/HCO (Folmer et al., 1994) or LepF1/LepR1 (Hebert et al., 2004).

**LCO**: 5’ GGT CAA CAA ATC ATA AAG ATA TTG G 3’

**HCO**: 5’ TAA ACT TCA GGG TGA CCA AAA AAT CA 3’

**LepF1**: 5’ ATT CAA CCA ATC ATA AAG ATA TTG G 3’

**LepR1**: 5’ TAA ACT TCT GGA TGT CCA AAA AAT CA 3’

Both primer sets used the following PCR recipe and thermal conditions:

| **PCR mix:** |  |
| --- | --- |
| **Reagents** | **Volume (μl)** |
| MilliQ water | 13.6 |
| 10x PCR Buffer | 2 |
| BSA (10mg/ml) | 2 |
| MgCl_2_ (50mM) | 0.8 |
| primer LCO or LepF1 (20μM) | 0.2 |
| primer HCO or LepR1 (20μM) | 0.2 |
| dNTPs (each 25mM) | 0.1 |
| *Taq* (Bioline 5U/μl) | 0.1 |
| DNA template | 1.0 |
| **TOTAL** | **20** |
|  |  |
| **PCR conditions:** |  |
| Step 1: 94°C | 2 minutes |
| Step 2: 94°C | 30 seconds |
| Step 3: 51°C | 30 seconds |
| Step 4: 72°C | 40 seconds |
| Go to step 2, repeat 34x |  |
| Step 5: 72°C | 5 minutes |
| Step 6: 10°C | hold |
| END |  |

**2. Internal transcribed spacer region 2 (ITS2)**

Primers used are ITS2f/ITS2r, following Campbell et al. (1993).

**ITS2f**: 5’ TGT GAA CTG CAG GAC ACA TG 3’

**ITS2r**: 5’ AAT GCT TAA ATT TAG GGG GTA 3’

| **PCR mix:** |  |
| --- | --- |
| **Reagents** | **Volume (μl)** |
| MilliQ water | 13.74 |
| 10x PCR Buffer | 2 |
| BSA (10mg/ml) | 2 |
| MgCl_2_ (50mM) | 0.6 |
| primer ITS2f (20μM) | 0.2 |
| primer ITS2r (20μM) | 0.2 |
| dNTPs (each 25mM) | 0.16 |
| *Taq* (Bioline 5U/μl) | 0.1 |
| DNA template | 1.0 |
| **TOTAL** | **20** |
|  |  |
| **PCR conditions:** |  |
| Step 1: 94°C | 2 minutes |
| Step 2: 94°C | 30 seconds |
| Step 3: 52°C | 40 seconds |
| Step 4: 72°C | 40 seconds |
| Go to step 2, repeat 34x |  |
| Step 5: 72°C | 5 minutes |
| Step 6: 10°C | hold |
| END |  |

**3. Phosphogluconate dehydrogenase (PGD)**

Primers used are PGD_hym_3F/PGD_hym_intRb, following Malm and Nyman (2015).

**PGD_hym_3F**: 5’ TGG TRC ACA AYG GMA THG ART AYG G 3’

**PGD_hym_intRb**: 5’ ATR ATR CAN CCD CCY CKC CAC AT 3’

| **PCR mix:** |  |
| --- | --- |
| **Reagents** | **Volume (μl)** |
| MilliQ water | 13.54 |
| 10x PCR Buffer | 2 |
| BSA (10mg/ml) | 2 |
| MgCl_2_ (50mM) | 0.8 |
| primer PGD_hym_3F (20μM) | 0.2 |
| primer PGD_hym_intRb (20μM) | 0.2 |
| dNTPs (each 25mM) | 0.16 |
| *Taq* (Bioline 5U/μl) | 0.1 |
| DNA template | 1.0 |
| **TOTAL** | **20** |
|  |  |
| **PCR conditions:** |  |
| Step 1: 94°C | 2 minutes |
| Step 2: 94°C | 30 seconds |
| Step 3: 60°C | 40 seconds |
| Step 4: 72°C | 40 seconds |
| Go to step 2, repeat 34x |  |
| Step 5: 72°C | 5 minutes |
| Step 6: 10°C | hold |
| END |  |

**4. wingless (wg)**

Some samples were amplified using the primer set beewgFor/Lepwg2a, following Danforth et al. (2004; for beewgFor) and Brower and DeSalle (1998; for Lepwg2a).

Most samples were amplified using custom primers, sfwgF/sfwgR, designed during this study for use on argid sawflies.

**beewgFor**: 5’ TGC ACN GTS AAG ACC TGY TGG ATG AG 3’

**Lepwg2a**: 5’ ACT ICG CAR CAC CAR TGG AAT GTR CA 3’

**sfwgF**: 5’ AAC CTS AAG GAC CGH TTC GAC 3’

**sfwgR**: 5’ CTC GAC SAC SAC SAC CTC 3’

Conditions for beewgFor/Lepwg2a were:

| **PCR mix:** |  |
| --- | --- |
| **Reagents** | **Volume (μl)** |
| MilliQ water | 13.3 |
| 10x PCR Buffer | 2 |
| BSA (10mg/ml) | 2 |
| MgCl_2_ (50mM) | 1.0 |
| primer beewgFor (20μM) | 0.2 |
| primer Lepwg2a (20μM) | 0.2 |
| dNTPs (each 25mM) | 0.2 |
| *Taq* (Bioline 5U/μl) | 0.1 |
| DNA template | 1.0 |
| **TOTAL** | **20** |
|  |  |
| **PCR conditions:** |  |
| Step 1: 94°C | 2 minutes |
| Step 2: 94°C | 30 seconds |
| Step 3: 54°C | 40 seconds |
| Step 4: 72°C | 40 seconds |
| Go to step 2, repeat 34x |  |
| Step 5: 72°C | 5 minutes |
| Step 6: 10°C | hold |
| END |  |

Conditions for sfwgF/sfwgR were:

| **PCR mix:** |  |
| --- | --- |
| **Reagents** | **Volume (μl)** |
| MilliQ water | 13.54 |
| 10x PCR Buffer | 2 |
| BSA (10mg/ml) | 2 |
| MgCl_2_ (50mM) | 0.8 |
| primer sfwgF (20μM) | 0.2 |
| primer sfwgR (20μM) | 0.2 |
| dNTPs (each 25mM) | 0.16 |
| *Taq* (Bioline 5U/μl) | 0.1 |
| DNA template | 1.0 |
| **TOTAL** | **20** |
|  |  |
| **PCR conditions:** |  |
| Step 1: 94°C | 2 minutes |
| Step 2: 94°C | 30 seconds |
| Step 3: 61°C | 40 seconds |
| Step 4: 72°C | 40 seconds |
| Go to step 2, repeat 34x |  |
| Step 5: 72°C | 5 minutes |
| Step 6: 10°C | hold |
| END |  |

**References**

Brower, A.V.Z., and DeSalle, R. (1998). Patterns of mitochondrial versus nuclear DNA sequence divergence among nymphalid butterflies: the utility of wingless as a source of characters for phylogenetic inference. Insect Mol. Biol. **7**, 73-82.

Campbell, B.C., Steffen-Campbell, J.D., and Werren, J.H. (1993). Phylogeny of the *Nasonia* species complex (Hymenoptera: Pteromalidae) inferred from an internal transcribed spacer (ITS2) and 28S rDNA sequences. Insect Mol. Biol. 2, 225-237.

Danforth, B.N., Brady, S.G., Sipes, S.D., and Pearson, A. (2004). Single-copy nuclear genes recover Cretaceous-age divergences in bees. Syst. Biol. 53, 309-326.

Folmer, O., Black, M., Hoeh, W., Lutz, R., and Vrijenhoek, R. (1994). DNA primers for amplification of mitochondrial cytochrome *c* oxidase subunit I from diverse metazoan invertebrates. Mol. Mar. Biol. Biotech. 3, 294-299.

Hebert, P.D.N., Penton, E.H., Burns, J.M., Janzen, D.H., and Hallwachs, W. (2004). Ten species in one: DNA barcoding reveals cryptic species in the neotropical skipper butterfly *Astraptes* *fulgerator*. Proc. Nat. Acad. Sci. U.S.A. 101, 14812-14817.

Malm, T., and Nyman, T. (2015). Phylogeny of the symphytan grade of Hymenoptera: new pieces into the old jigsaw(fly) puzzle. Cladistics. 31, 1-17.
